# Sequence-Dependent Shape and Stiffness of DNA and RNA Double Helices: Hexanucleotide Scale and Beyond

**DOI:** 10.1021/acs.jcim.5c00576

**Published:** 2025-08-25

**Authors:** Pavlína Slavníková, Marek Cuker, Eva Matoušková, Ivan Čmelo, Marie Zgarbová, Petr Jurečka, Filip Lankaš

**Affiliations:** † Department of Informatics and Chemistry, 52735 University of Chemistry and Technology Prague, 166 28 Prague, Czech Republic; ‡ CZ-OPENSCREEN: National Infrastructure for Chemical Biology, Faculty of Chemical Technology, University of Chemistry and Technology Prague, 166 28 Prague, Czech Republic; § Molecular Design Group, School of Chemical Sciences, Dublin City University, D09 V209 Glasnevin, Ireland; ∥ Department of Physical Chemistry, Faculty of Science, Palacký University, 771 46 Olomouc, Czech Republic

## Abstract

The structure and deformability of double-stranded DNA and RNA depend on the sequence of bases, affecting biological processes and nanostructure design, but this dependence is incompletely understood. Here we present mechanical properties of DNA and RNA duplexes inferred from atomic-resolution, explicit-solvent molecular dynamics (MD) simulations of 107 DNA and 107 RNA oligomers containing all hexanucleotide sequences. In addition to the level of rigid bases, minor and major grooves, we probe the length and sequence dependence of global material constants such as persistence lengths, stretching and twisting rigidities. We propose a simple model to predict sequence-dependent shape and nonlocal, harmonic stiffness for an arbitrary sequence, validate it on an independent set of MD simulations for DNA and RNA duplexes containing all pentamers, and demonstrate its utility in various applications. The large amount of the simulated data enabled us to study rare events, such as base-pair opening, or flips of the A-RNA sugar pucker into the B domain and the related dynamics of the 2′-OH group. Together, this work provides a comprehensive sequence-specific description of DNA and RNA duplex mechanics, forming a baseline for further research and allowing for a broad range of applications.

## Graphical abstract

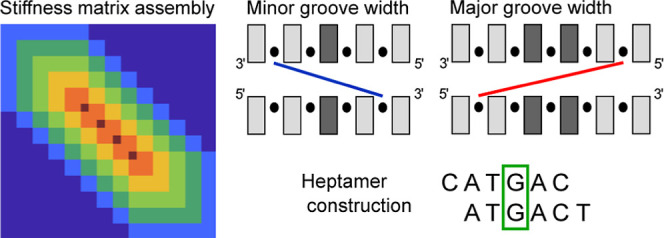

## Introduction

Double-stranded DNA (dsDNA) carries genetic information in all cellular life. RNA in biological contexts can take up a variety of structures, but its double helical form (dsRNA) is a prominent motif.^1^ Since the early days of DNA crystallography,^2^ a variety of experimental results have indicated that the three-dimensional shape and structural flexibility of dsDNA^3–7^ and dsRNA^8–12^ depend on the sequence of bases, affecting their interaction with proteins^13,14^ and small ligands.^15–18^ The sequence-specific structural information significantly enhances the accuracy of algorithms used for predicting transcription factor affinities^19–24^ and identifying promoter regions.^25,26^ Sequence-dependent mechanical properties affect DNA supercoiling,^27^ nucleosome positioning,^28–31^ and chromatin structure,^32^ while the exact shape and stiffness of RNA helices influence the structural dynamics and tertiary contacts in the ribosome and other assemblies.^11,33,34^ Both dsDNA and dsRNA are also critical components in construction of artificial NA nanostructures.^35–38^ It is therefore important to understand how the three-dimensional structure and deformability of DNA and RNA double helices depend on their sequence of bases.

The sequence-dependent shape and stiffness of NA duplexes can be described at various levels of detail, as reviewed.^33,39–45^ A widely used approach is to model NA bases as rigid objects. Their relative displacement and rotation are then defined by the intra-basepair coordinates shear, stretch, stagger, buckle, propeller and opening, as well as inter-basepair (or step) coordinates shift, slide, rise, tilt, roll and twist.^46–49^ Collectively we will call them rigid-base coordinates.

Initial descriptions treated the DNA duplex as a chain of independent base-pair steps^3,50^ and independent base pairs.^51^ More recent models of this type, parametrized from molecular dynamics (MD) simulations or crystallographic data, still focus on the individual base-pair steps, but the model parameters now depend on the tetrameric sequence context. This approach, initiated by the ABC consortium,^52,53^ provided a set of rules relating DNA structure and deformability to the sequence^54,55^ as well as to the backbone conformation^56^ and led to the formulation of a local, multistate model of DNA shape and stiffness.^57^

The assumption of independent pairs and steps was soon challenged by the discovery of structural correlations along the helix,^47,50^ which have been extensively studied.^58–62^ The work^63^ introduced a sequence-specific, nonlocal stiffness matrix, enabling one to probe the elastic couplings along a particular DNA oligomer and their sequence variations. The stiffness matrix turned out to be close to banded, with the most significant entries within overlapping dimeric blocks (two base pairs and the step in between, 18 rigid-base coordinates in total).

This finding inspired the development of a nonlocal, sequence-dependent rigid-base model of DNA^64^ and its software implementation, cgDNA.^65^ To deduce the sequence-dependent shape, the algorithm entails the inversion of a banded, but not block diagonal, matrix. Since the inverse of a banded (but not block diagonal) matrix is in general dense, the (relatively few) parameters in the banded matrix enable one to predict long-range sequence-context effects on the shape. The stiffness matrix, by contrast, is assembled from dimeric blocks and therefore covers only the dinucleotide dependence, which is certainly an oversimplification.^44^ The latest version of the model, cgNA+,^66^ is still based on dinucleotide blocks but the description is now more detailed, as not only the bases, but also the phosphate groups, are modeled as rigid bodies. If the phosphate group coordinates are marginalized (integrated out), the resulting, rigid-base-only stiffness matrix is again dense and therefore the model can in principle predict the sequence-dependent, rigid-base elastic constants beyond the dinucleotide level. Indeed, the model reproduces sequence-averaged elastic couplings much better that the original cgDNA.^62^ Whether also the sequence-dependent elasticity is captured well remains to be seen. Furthermore, although the phosphates are explicitly present, to deduce sequence-dependent width and stiffness of major and minor grooves, a decisive factor in protein and ligand binding, from the model would not be straightforward.

In an important development, a nonlocal, multistate description of DNA based on an Ising-type model^67^ has been proposed. The model exhibits tetrameric sequence dependence of the shape, the stiffness matrix is assembled from tetrameric blocks. A later Ising-type description^68^ considers two subsequent base-pair steps embedded in a pentameric sequence. The banded stiffness matrix, therefore, consists of overlapping trimeric blocks (involving step coordinates only), while the blocks parametrically depend on the embedding pentamer.

A different approach has been introduced in ref 19, which reported DNA simulations covering all pentanucleotide sequences in many different contexts. The extensive sequence coverage was made possible by a highly simplified underlying physical model, describing the DNA duplex by selected collective and internal variables and using an implicit solvent model.^69,70^ Based on the simulated data, tools have been designed and applied to analyze structural profiles of transcription binding sites^19,21,71–74^ and to identify shape motifs in genomes.^23,75^ The pentameric data include the shape parameters (equilibrium rigid base coordinates and minor groove widths) and their flexibility (standard deviations). Recently, a deep learning approach has been developed to estimate sequence context effects beyond the simulated pentameric data.^22^ However, modeling nucleic acids in implicit solvent presents considerable limitations.^76^ The model also does not cover RNA duplexes and, importantly, does not provide the stiffness matrix, necessary to compute the full elastic energy of a deformed structure.

Taken together, existing models of sequence-dependent shape and stiffness of NA duplexes all exhibit substantial limitations. Some are confined to a fixed, rather narrow sequence range.^67,68^ Others have overcome (at least partially) this constraint,^22,66^ but they are complex models parametrized in a highly nontrivial manner and intended to be used essentially as a black box. Thus, there is a pressing need for a straightforward, transparent, essentially model-free approach to predict sequence-specific shape and mechanical properties of nucleic acid duplexes.

Here, we take a step forward in this direction. We present atomic-resolution, explicit-solvent MD simulations and analysis of DNA and RNA duplexes involving all hexanucleotide sequences, compacted into a minimal set of 107 DNA and 107 RNA oligomers, each 33 base pairs (bp) long. Based on the MD data, we reveal the pentameric scale as the minimum range of elastic couplings between rigid bases. The hexanucleotide scale covers this range, involves pentamers in many different contexts and, importantly, enables us to also study the sequence-dependent width and stiffness of the major groove, inaccessible at the pentameric level. We describe hexanucleotide context effects on base-pair step and major groove structure and flexibility, and heptameric context effects on base-pair and minor groove structure and flexibility, where the heptameric properties are estimated from overlapping hexamers. Furthermore, we propose a simple method to construct a sequence-specific, rigid-base stiffness matrix from the hexameric data. In addition to the rigid-base level, we study global material constants (bending persistence length, stretch modulus, twist stiffness, etc.). The large, well-balanced set of the simulated oligomers allowed us to examine how these constants depend on the duplex length and on its sequence, greatly extending the limited knowledge available so far.

To further validate our predictions, we produced an independent MD set of 52 DNA and 52 RNA oligomers involving all pentameric sequences. The validation indicates excellent predictive power of the model, corroborated by a number of experiment-oriented applications we present. Finally, the sheer amount of the simulated data allowed us to detect rare events, such as base-pair opening dynamics or transient flips of the A-RNA sugar pucker into the B domain and the related motion of the 2′-OH group.

## Methods

### Sequence Design

We designed a 516 base-pair (bp) minimal sequence containing each unique pentamer (512 in total) exactly once. To do this, a basic depth-first search approach was developed (Supporting Information Methods). The script took seconds to run on a desktop PC and is available at https://zenodo.org/records/15575888.

The script was also tested for other *k*-mer sizes, from dimers to nonamers. For odd-numbered *k*-mers, the method seems to perform well, quickly producing optimal sequences. However, this approach performs significantly worse for even-numbered *k*-mers, likely due to their possible self-complementarity, resulting in a less favorable search tree topology. Thus, to obtain the hexameric sequence, the ShortCAKE tool by Orenstein and Shamir^77^ was used, as it provides optimal (shortest possible) sequences both for odd- and even-numbered *k*-mers.

The optimal hexameric sequence was partitioned into 107 duplex oligomers (set107), each 25 bp long, the minimal pentameric sequence was split into 52 duplexes, each 14 bp long (set52). Each sequence was capped by a GCGC tetramer at the ends to isolate possible end fraying.^78^ The same sequence set (with thymines mutated to uracils) was used for the RNA duplexes. In set107, the overwhelming majority of hexamers (3912 out of 4096) are present exactly once, and 184 exactly twice. The partitioning into 107 sequences was found optimal to balance the number of systems to be simulated and the simulation speed. The set107 sequences are listed in a separate Supporting Information file, the set52 sequences are in Tables S1 and S2.

A set of 14 duplexes (set14) covering all tetrameric sequences, each 22 bp long (14 bp plus the GCGC caps, Table S3) was used to find the optimal cutoff for our stiffness matrix model (see below). The MD data for the DNA version were taken from an earlier publication,^56^ the RNA version was simulated for this study. To examine the effective range of base–base interactions in dsDNA and dsRNA, we also employed the MD data for a 33 bp oligomer in its DNA and RNA version (here denoted s0) from our previous work.^79^ Additionally, the DNA and RNA forms of s0 with neutralized phosphates and no ions, as well as those with neutralized phosphates and added 150 mM KCl, were simulated.

### MD Simulations and Analysis

Atomic-resolution, explicit-solvent, unrestrained MD simulations were performed using the Amber 22 suite of programs. Each oligomer was built in its B-DNA or A-RNA form by the *nab* module of Amber and solvated with SPC/E waters in a truncated octahedron periodic box. K^+^ counterions were added to compensate for the NA charge, additional K^+^ and Cl^–^ ions were included to mimic the physiological concentration of 150 mM KCl. For the DNA oligomers, the OL15 force field^80^ was used which, together with parmbsc1 ^81^ and more recent parametrizations tumuc1,^82^ OL21 ^83^ and OL24,^84^ belongs to the family of recent Amber force fields and is considered to reliably represent the structure and dynamics of B-DNA.^85–87^ The well-established χOL3 force field^88–90^ was employed for RNA, the ions were parametrized according to Dang.^91^ To produce the DNA and RNA neutral oligomers of the sequence s0, the +1 charge was split equally over the nonbridging O1P and O2P oxygens in both strands, since this is the location of the negative charge due to the proton release in solution.^92^

The systems were equilibrated by the standard ABC protocol,^53^ followed by a production run of 2 μs for each DNA sequence from the hexameric set107, and 1 μs for all the other systems, at 300 K and 1 atm. Hydrogen mass repartitioning^93^ and a 4 fs time step were used for the production, snapshots were recorded every 10 ps. The *cpptraj* module of Amber^94^ was employed to extract intra-basepair coordinates (shear, stretch, stagger, buckle propeller and opening), as well as base-pair step (shift, slide, rise, tilt, roll, twist) and helical coordinates (X-disp, Y-disp, helical rise, tip, inclination and helical twist) as defined by the 3DNA algorithm.^48^ Watson–Crick hydrogen bond lengths, and distances between phosphorus atoms used to define the groove widths, were also extracted with *cpptraj*. Altogether, this study covers all-atom, explicit-solvent, unrestrained MD simulations of 350 NA oligomers, the total simulation time is 0.46 ms.

#### Trajectory Filtering

Snapshots with at least one hydrogen bond (H-bond) broken anywhere within the central part (25 or 14 bp plus the flanking inner GC) were excluded from the analysis. We consider a H-bond broken if the distance between its heavy atoms exceeds 4 Å, as in 3DNA.^48^ Furthermore, we manually checked the plots of all the coordinates and excluded the (very few) parts with obvious anomalies, corresponding to noncanonical structures and discussed below. In this way, at least 89% of snapshots in a DNA trajectory and at least 67% in an RNA trajectory were left for further processing. The relatively high fraction of snapshots filtered out is due in part to the rather strict filtration criteria, i.e. that we exclude snapshots with a broken H-bond anywhere in the duplex, except the two outer G-C pairs. The low minimal yield for RNA is due to one single sequence, where a 250 ns flip of one sugar into the B-form induced large structural perturbations and the corresponding part of the trajectory was filtered out (Figure 9). The average fraction of snapshots kept is 95% for DNA and 86% for RNA duplexes. Of note, three of the RNA sequences exhibited a H-bond break within the first 100 ns which was not repaired until the end of the simulation. These sequences were resimulated using a different initial velocity seed, and the long-living break never appeared again.

**Figure 1:**
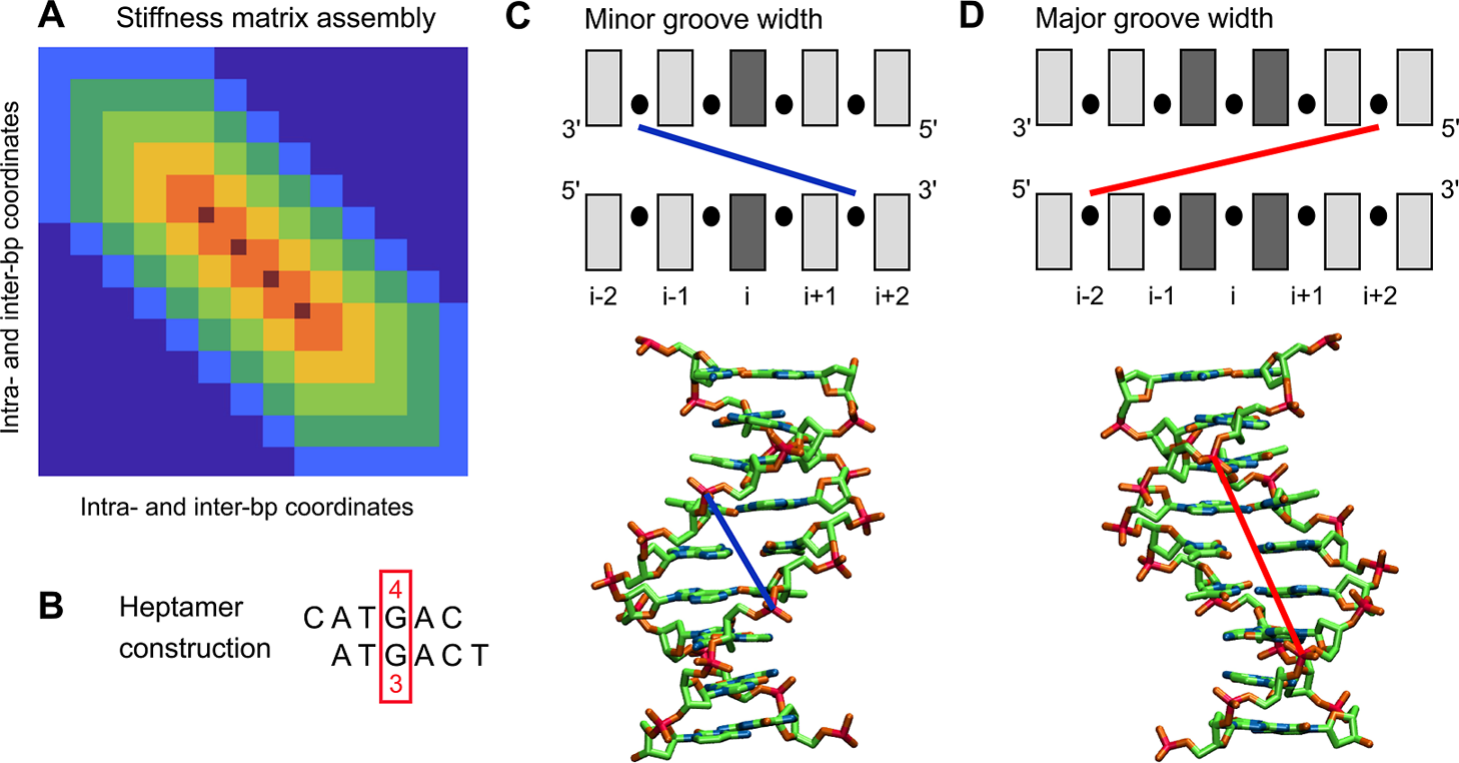
(A) The stiffness matrix construction for an arbitrary sequence. The sequence is divided into overlapping hexamers, and the precomputed hexameric blocks are assembled as shown. Each block of dimension 66 × 66 involves intra-basepair and step coordinates of the given hexamer, ordered in a natural way (first pair, first step, second pair, etc.) The entries in the overlapping parts are arithmetically averaged. The blocks exhibit multiple overlaps (zero to five marked by cyan, dark green, green, orange, red and dark red, respectively). The assembled matrix is zero outside the hexameric band (dark blue). (B) A heptamer constructed from overlapping hexamers. In this example, the heptamer CATGACT consists of two hexamers, CATGAC and ATGACT. The model parameters for the heptamer’s central pair are computed as arithmetic means of the values in the two hexamers (4th and 3rd pair, respectively). (C) The minor groove width is defined as the distance between phosphorus atom centers at the 3′ sides of a pentamer in its heptameric context, and is assigned to the central pair. (D) The major groove width is the distance between P atom centers at the 5′ sides of a hexamer, and is assigned to its central step.

**Figure 2:**
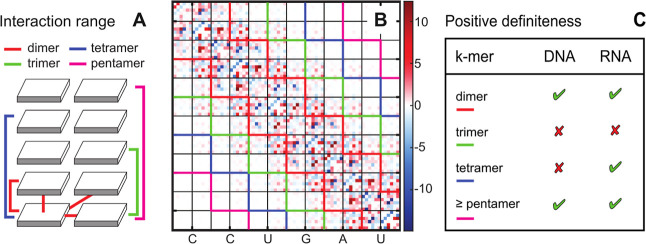
Effective base–base interactions in a double helix. (A) Schematic representation of base–base interactions in a duplex. (B) The stiffness matrix for the s0 RNA sequence shown as an example, with bands indicating different base–base interaction ranges, color coding as in (A). The stiffness matrix was computed from the covariance matrix of the intra-basepair and step coordinates using eq 3. The 6 × 6 blocks corresponding to the coordinates of a given pair, and those of the following step, are delineated in black. Only the central part of the matrix is shown. (C) Definiteness of stiffness matrices where the interaction ranges indicated are imposed (entries outside the band are set to zero). The data in (C) apply to all the DNA and RNA duplexes examined in this work, including the neutralized ones (with and without added ions).

**Figure 3:**
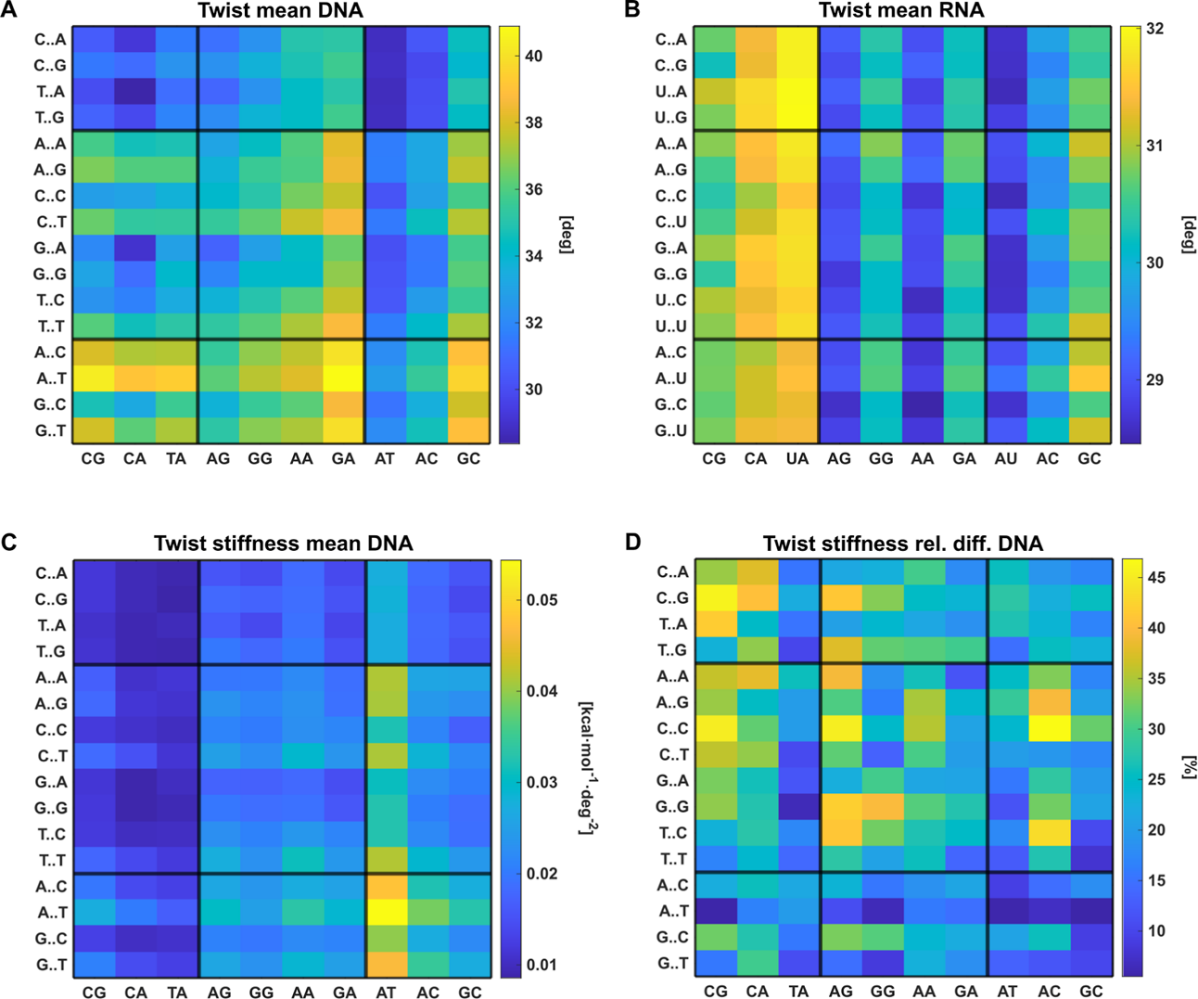
DNA (A) and RNA (B) twist for the central step of the tetrameric sequences, each averaged over all its hexameric contexts. (C) Tetramer-specific DNA twist stiffness. (D) The relative variability of DNA twist stiffness over the hexameric contexts. It is computed as the difference between the maximum and minimum hexameric value for the given central tetramer, divided by the mean over all the hexameric contexts. The black lines separate the YR, RR, and RY sequence groups (R—purine, Y—pyrimidine). Analogous plots for the remaining coordinates are at https://zenodo.org/records/15575888.

**Figure 4:**
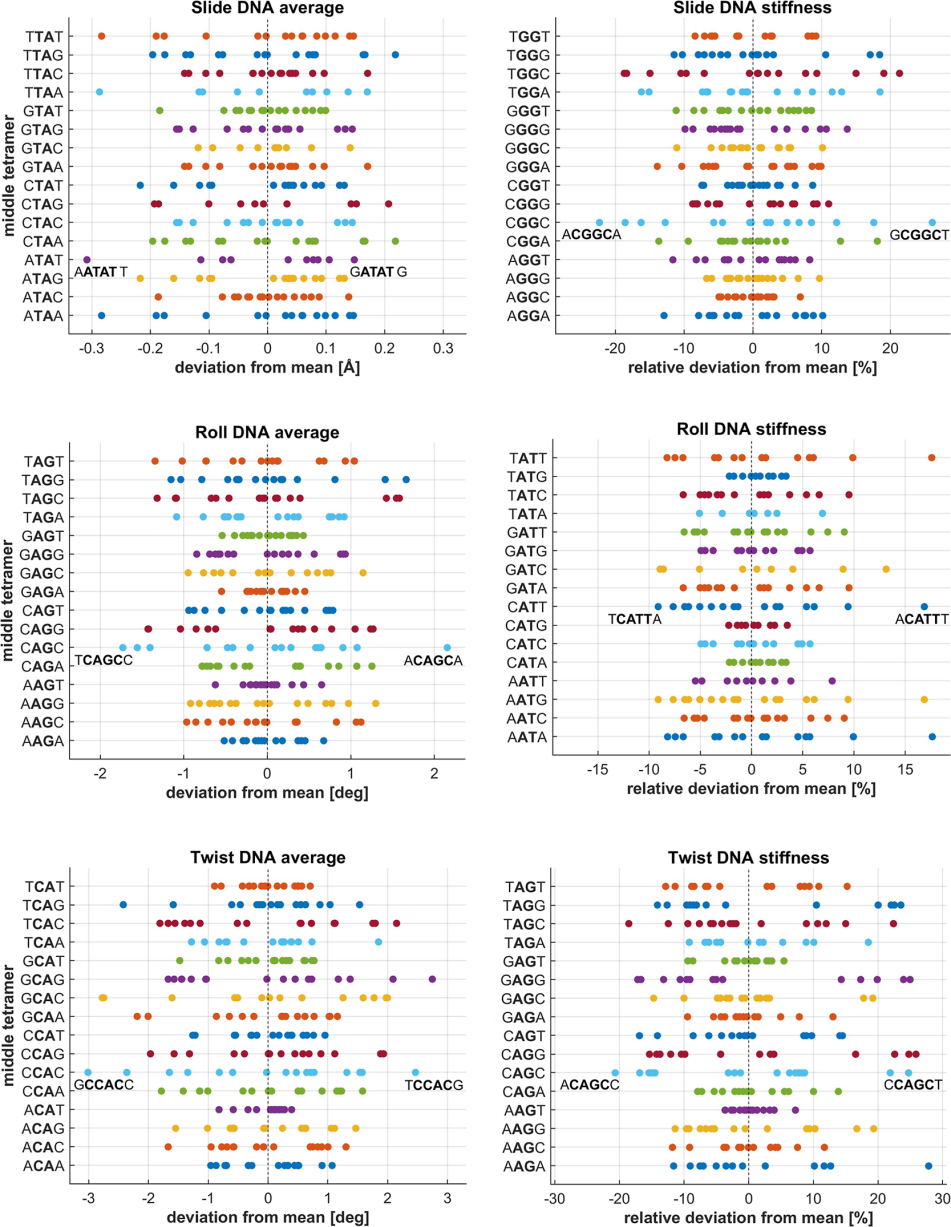
Hexameric context dependence for selected DNA tetramers. Each panel shows all tetramers sharing the same central step. The reference values are means over all hexameric contexts for the given tetramer. Hexamer-dependent deviations from these values are plotted.

**Figure 5:**
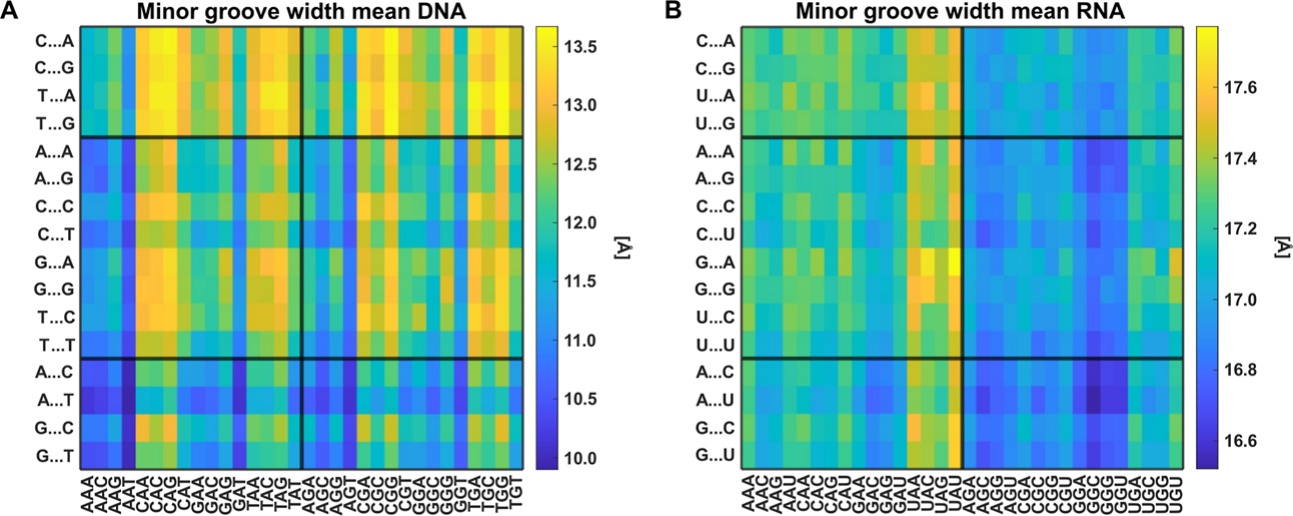
Sequence-dependent variability of the minor groove width. Values for pentameric sequences, each averaged over all its heptameric contexts, for dsDNA (A) and dsRNA (B) are shown.

**Figure 6:**
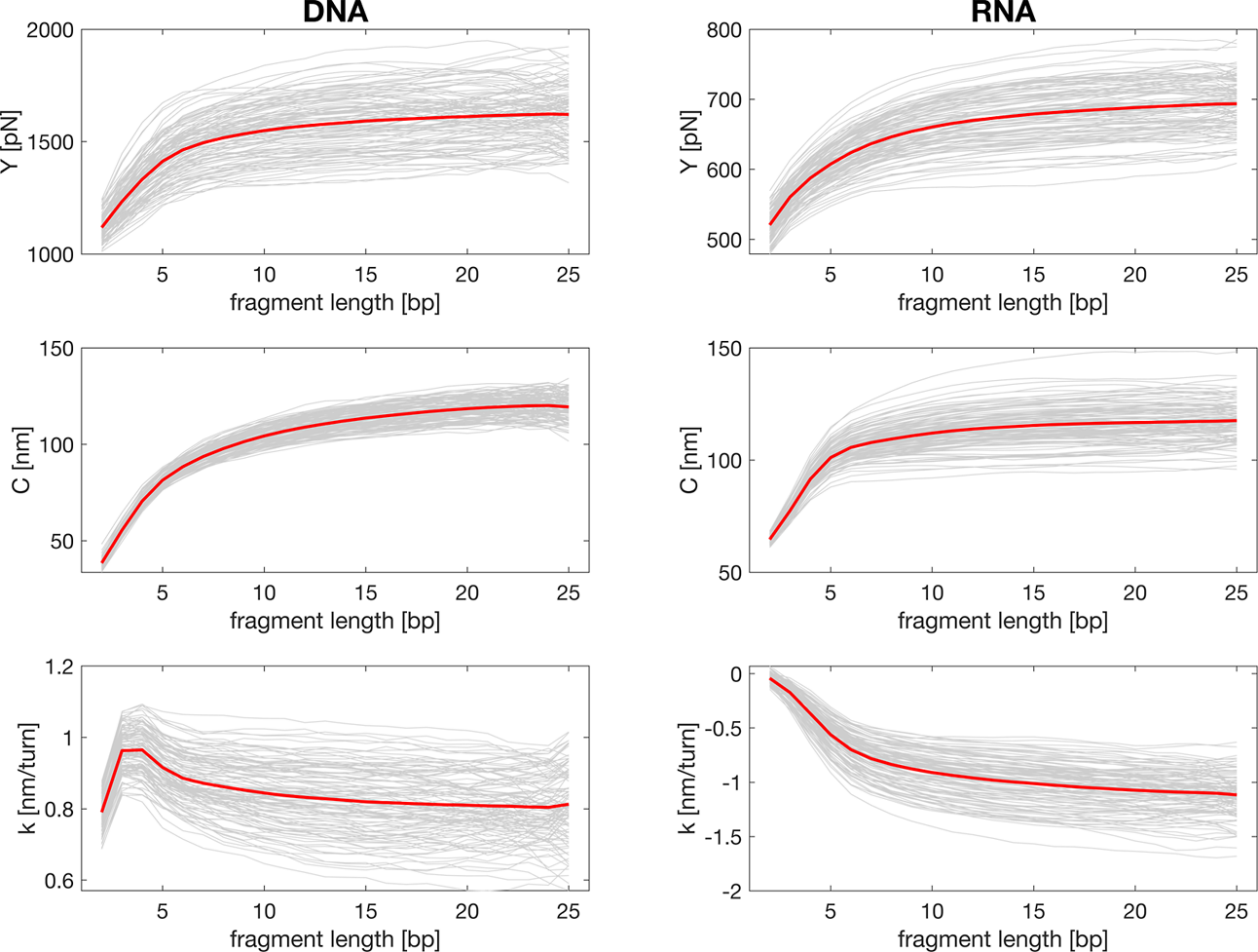
Length-scale dependence of the elastic constants. For each of the simulated 107 DNA and 107 RNA duplexes, the constants of the central 25 bp part and all its subsequences (fragments, 2–25 bp) were calculated. Values for fragments of the same length were then averaged, resulting in one plotted line for each sequence (gray lines). The data were then further averaged over the sequences (red lines). The arithmetic mean was used in all cases.

**Figure 7:**
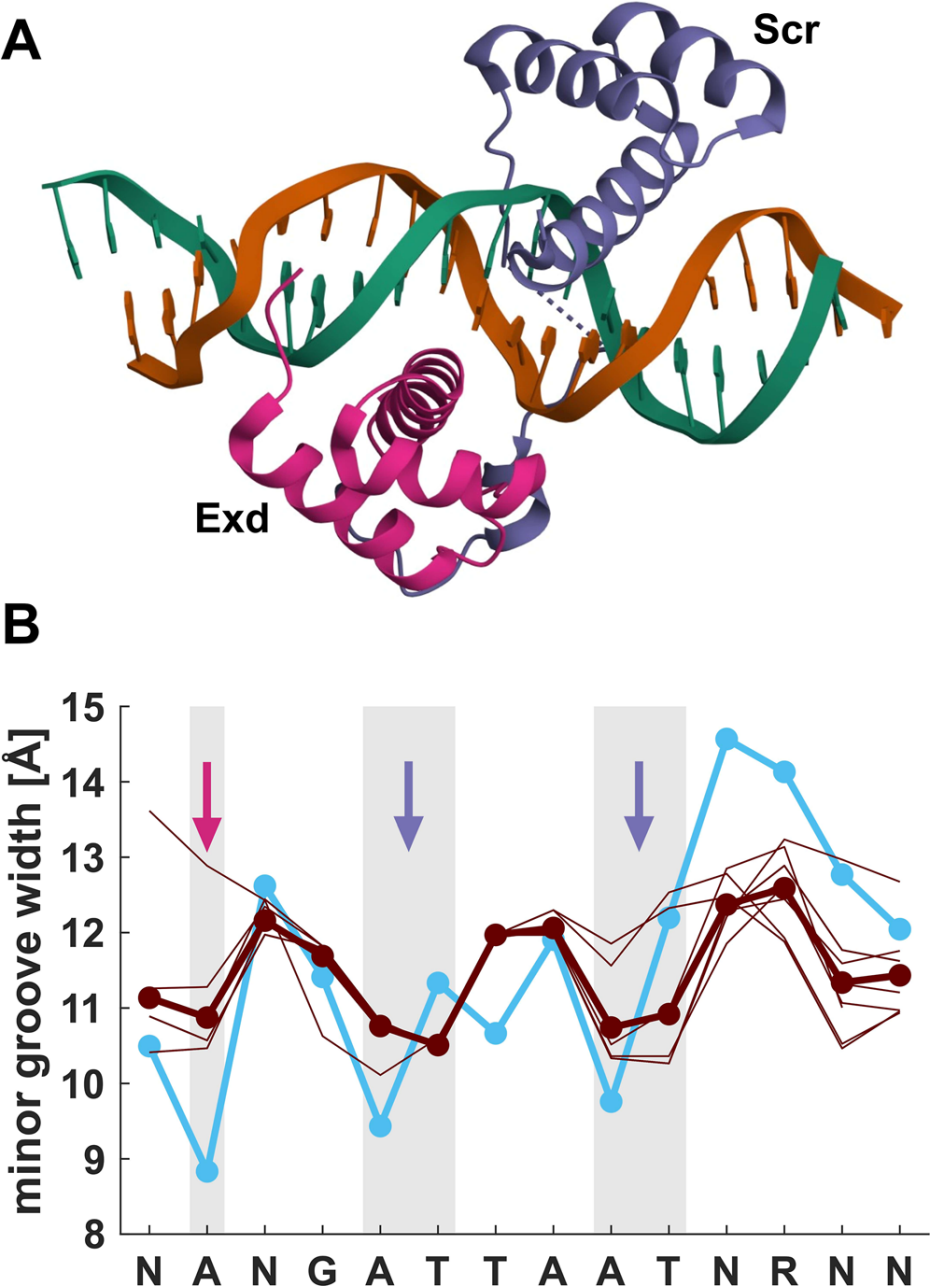
(A) The crystal structure of the Exd (purple) and Scr (blue) complex with DNA (2R5Z^146^). (B) Minor groove profiles for the Exd–Scr binding site. The profiles predicted for the top ten highest-affinity sequences^71^ (thin brown lines) and their average (brown, bold). The arrows indicate sites where the arginine residues from Exd (purple arrow) and Scr (blue arrows) protrude into the minor groove. The minor groove profile from the crystal structure of the complex (cyan) is also shown.

**Figure 8:**
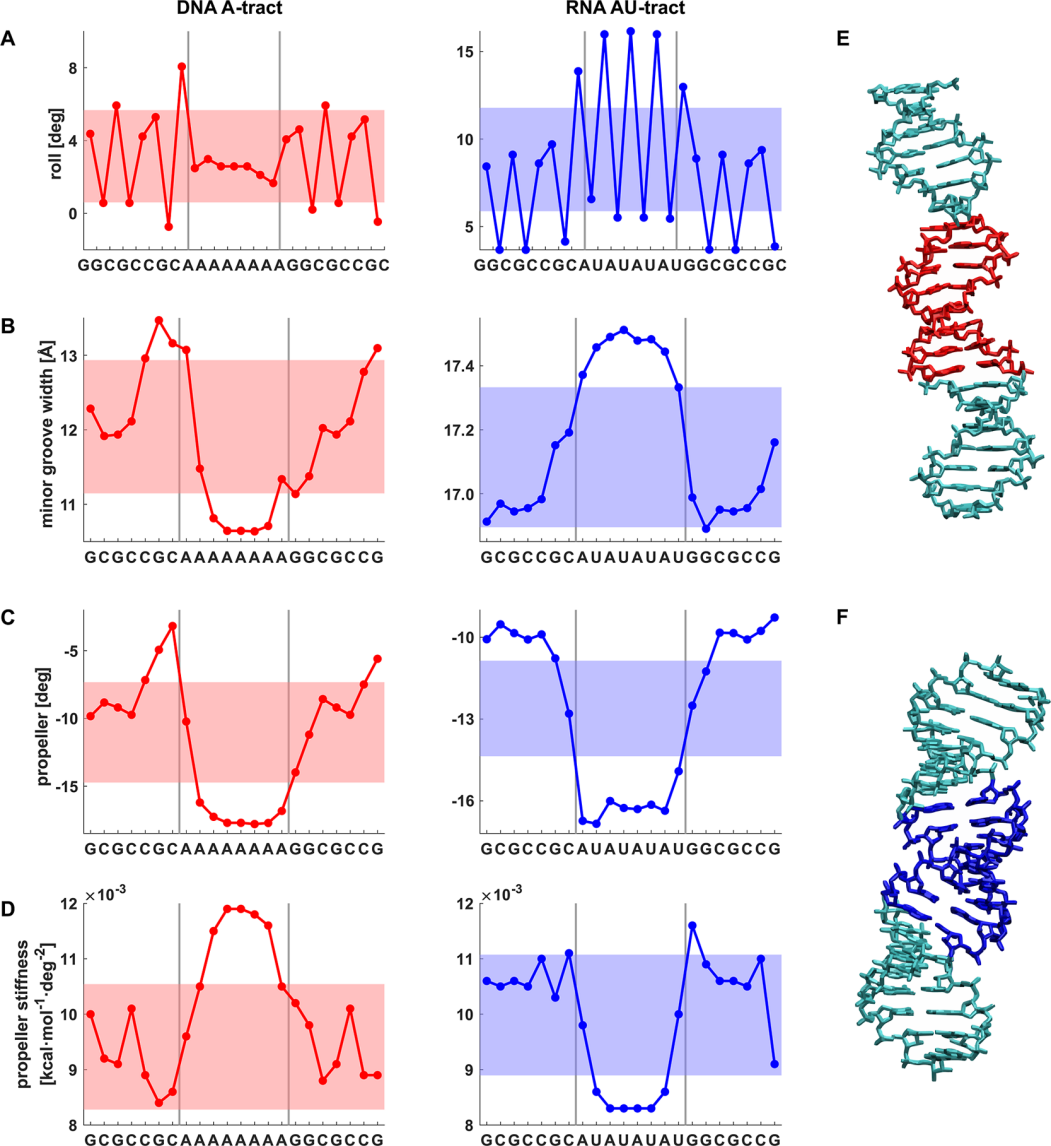
Profiles of sequence-dependent shape and stiffness parameters for DNA A-tract and RNA AU-tract sequences predicted by our model. The colored stripes indicate values within one standard deviation from the mean over all hexameric (roll) or heptameric sequences.

**Figure 9:**
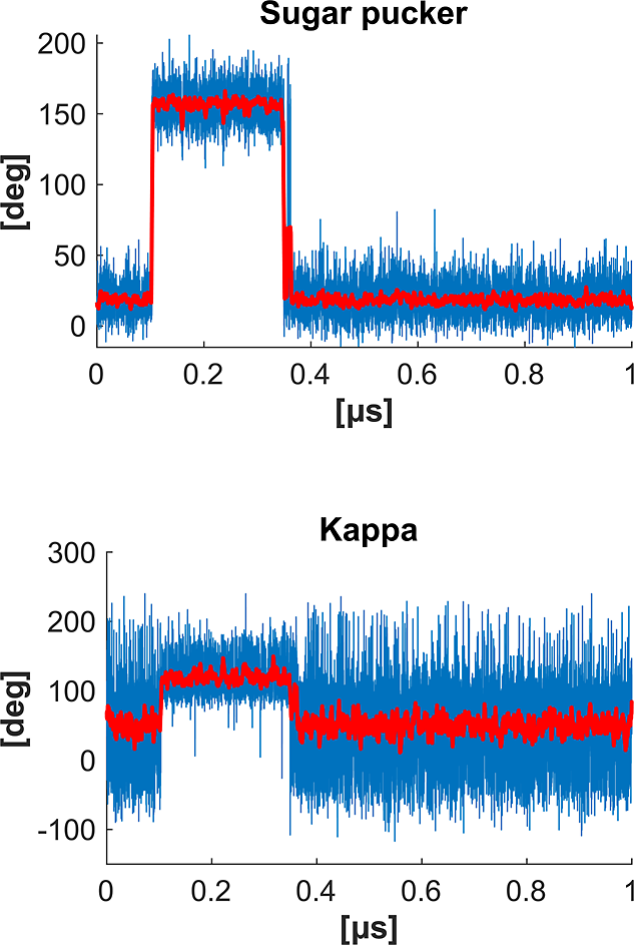
A long-living (250 ns) flip of the RNA sugar pucker into the B domain (RNA set107, seq 79, U in the uauUguu context), accompanied by a flip in the torsion angle κ involving the 2′-OH group. Data averaged over a sliding window of 3 ns are shown in red.

#### Groove Definitions

The minor groove width is defined here as the distance between the centers of the phosphorus (P) atoms at the 3′ ends of a pentamer, and assigned to the pentamer’s central pair (Figure 1C). This definition is based on the observation in^95^ that the distance is close to a local minimum of distances between the strands. The authors in ref 95 proposed to take the mean of two neighboring P–P distances, just to be able to assign the minor groove width to a step, a definition implemented also in 3DNA.^48^ However, taking just one distance suits better our purposes, since both P atoms are now inside a pentamer. Moreover, the definition employed here avoids artificial correlation between neighboring minor groove widths, which occurs in the mean-distance definition of^95^ just because the two neighboring widths share a common P–P distance. The major groove width is defined by the P–P distance between the 5′ ends of a hexamer and is assigned to its central step (Figure 1D), just as in.^48,95^

### Deformation Energy

The free energy cost of distorting the oligomer, or deformation energy *E*, at the rigid-base level is assumed in this work to be a quadratic function of the coordinate column vector **w**

1 $$E=\frac{1}{2}\left(w-ŵ\right)\cdot K\left(w-ŵ\right)$$

where **ŵ** is the (column) vector of equilibrium coordinates, given by the average of **w** over the canonical ensemble

2 ŵ=⟨w⟩

and **K** the stiffness matrix, related to the coordinate covariance matrix **Σ** as

3 $$K=k_{B}T\Sigma^{-1}$$

The ensemble means and covariances are estimated from the filtered MD trajectory.

The entries of the stiffness matrix are of three different physical dimensions: kcal/mol/Å^2^, kcal/mol/deg^2^ or kcal/mol/deg Å (eq 1). If we want to study properties of the stiffness matrix as a whole, such as its eigenvalues and eigenvectors, the matrix entries have to be made dimensionally uniform, or dimensionless. We choose the length scale of 1 Å. As for the angle scale, the value numerically equal to the twist density of the B-DNA or A-RNA helices is a standard choice in extensible elastic rod descriptions (e.g., refs 96–98 ). Taking the DNA value of 10.5 bp/turn^5,99,100^ and its 3.23 Å inter-bp distance,^101^ we obtain the helical pitch of 10.5 × 3.23 = 34 Å and the twist density 360/34 = 10.6 deg/Å, so that the angle scale for DNA would be 10.6°. For the RNA helix we have 11.3 bp/turn^2,102^ and 2.8 Å inter-bp distance,^103^ yielding the pitch of 31.6 ≈ 32 Å and the RNA angle scale 360/32 = 11.3°. Using a different line of reasoning, ref 104 proposed 1/5 rad, or 11.5°. Here, to make things simple, we choose 11° as the angle scale for both the DNA and RNA duplexes.

### Estimating Sequence-Dependent Shape and Flexibility

To define the equilibrium structure of a given base-pair step, we take the average coordinates over the (filtered) MD trajectory for the step in its hexameric context. The major groove width is inferred for the given hexamer and assigned to its central step. For the (very few) duplicate hexamers, the first one encountered in the sequence set (set107) is considered and the other one is ignored (but the variability between the duplicates is also examined, see below). From the unique hexamers, values for their complements are constructed, transforming the coordinates of the central step according to their parity: odd coordinates (shift, tilt, Y-disp, tip) change sign, the other, even ones (including major groove width) remain unchanged. Since the hexameric sequence dependence is assumed, the odd coordinates of the central step of self-complementary hexamers (e.g., ATATAT) are set to zero.

To define the equilibrium structure of a given base pair and minor groove width, we construct a heptamer by considering two overlapping hexamers, and compute the arithmetic mean of the values for the pair in the center (Figure 1B). Coordinate values for complementary heptamers are then constructed. The odd coordinates (shear, buckle) change sign, the even ones (including minor groove width) remain unchanged.

In addition to the shape, we also examine the coordinate stiffness (or flexibility), i.e. the stiffness constant *k*_*x*_ for deforming an individual coordinate *x* (intra-basepair or step coordinate, major or minor groove width), while all the other degrees of freedom in the duplex are unconstrained. It is computed as *k*_*x*_ = *k*_B_ *T*/var­(*x*), where the variance is estimated from the filtered MD trajectory. Notice that the stiffness constants *k*_*x*_ are even quantities.

The shape and stiffness of an arbitrary sequence is then estimated by applying a hexameric/heptameric sliding window. Thus, three pairs at each end are not covered. The shape and stiffness data in csv format for all the individual hexamers/heptamers are at https://zenodo.org/records/15575888.

### Estimating Sequence-Dependent Stiffness Matrix

To extract hexameric stiffness blocks from our MD data, we first compute, for the inner, hexamer-containing 25 bp part of each oligomer in set107, a stiffness matrix that is exactly zero outside a band defined by the overlapping hexameric blocks (Figure 1A). To this end, we use the maximum absolute entropy approach introduced in ref 105. By construction, the covariance related to this banded stiffness is equal to the original covariance matrix within the band (but may be different outside the band). The case considered in ref 105 only involved blocks with a single overlap (i.e., a matrix entry may belong to at most two different blocks), whereas in our case there are entries belonging to as many as six different blocks (dark red in Figure 1A). Nevertheless, the method applies to these multiple overlaps as well^106^ and is as follows. Each of the diagonal hexameric blocks of the original covariance matrix is inverted and written to the corresponding block in the stiffness matrix, with all entries in the overlapping regions summed up. Then the covariance blocks corresponding to overlaps between adjacent hexamers are inverted and subtracted from the corresponding blocks in the stiffness matrix. Finally, the stiffness matrix is multiplied by *k*_B_ *T*.

We apply this procedure to the simulated 107 dsDNA and 107 dsRNA oligomers to obtain the banded estimates of their stiffness matrices, then we cut out the hexameric blocks from them. We note in passing that even this (rather straightforward) procedure is nearly not necessary: the validation (see below) indicates only slightly worse results if the hexameric blocks are directly cut out from the raw stiffness matrices.

The coordinates involved in each hexameric block are ordered in a natural way, i.e. first pair, first step, second pair, second step, ..., last pair. For a pair, the ordering in as follows: shear, stretch, stagger, buckle, propeller, and opening. For a step we have shift, slide, rise, tilt, roll, and twist.

To obtain stiffness blocks for complementary hexamers, the entries have to be rearranged (the last pair is now the first one etc.) Moreover, the entries corresponding to odd–even combinations (e.g., tilt-twist) change sign.^64^ The odd–even entries for self-complementary hexamers are set to zero. To estimate the stiffness matrix of an arbitrary sequence, we assemble it from the hexameric blocks (Figure 1A). Entries in the overlapping parts are arithmetic means of entries in the individual blocks. By construction, the banded stiffness matrices of the sequences from which the blocks were cut out are reproduced exactly. The arithmetic averaging is certainly not the only possible: the overlapping parts are positive definite and various methods for averaging positive definite matrices have been proposed.^107^ We also tested the harmonic average (i.e., arithmetic average of the inverses), with nearly identical results. The individual hexameric blocks in csv format are at https://zenodo.org/records/15575888.

### Global Elasticity

Besides the rigid-base level, we also investigate elastic properties of the DNA and RNA duplexes at the global level, modeling them as extensible, bendable, and twistable elastic rods. The global length *l* and global twist ω of an oligomer or its part (fragment) are defined as the sum of helical rises and the sum of helical twists (in radians), respectively, as proposed in ref 108.

To compute the bending persistence length (p.l.) of a given oligomer, we examine directional correlations between its base-pair normals. The dynamic bending p.l. *l*_d_ is obtained by factoring out the static structure the way proposed in,^109^ with an additional prefactor α_d_, and is defined by the relation

4 $$\frac{\left\langle t_{i}\cdot t_{0}\right\rangle }{{\mathrm{t̂}}_{i}\cdot {\mathrm{t̂}}_{0}}=\alpha_{d}\exp\left(-\frac{l_{i0}}{l_{d}}\right)$$

where **t**_*i*_ and **t**_0_ are instantaneous normals of base pairs 0 and *i*, ${\mathrm{t̂}}_{i}$ and ${\mathrm{t̂}}_{0}$ are normals of the same pairs in the equilibrium structure (here reconstructed from hexamer-specific values of equilibrium base-pair step coordinates), the brackets denote average over the canonical ensemble, and *l*_*i*0_ is the ensemble average of the distance *l*_*i*_ (sum of helical rises) between the pairs 0 and *i*, *l*_*i*0_ = ⟨*l*_*i*_⟩. In the computation, base pair 0 is the first pair of the inner 25 bp hexamer-containing part (i.e., bp 5 of the whole sequence), and the index *i* increases from 1 to 24. The ensemble averages are estimated by averages over the filtered MD trajectory. The values of *l*_d_ and α_d_ are then deduced from fitting a straight line to the logarithm of the left-hand side as a function of *l*_*i*0_ (semilog plot). The prefactor α_d_ accounts for the possibility that the fitting line does not pass through the origin.^44,110^

The twist persistence length (p.l.) *l*_tw_ probed here reflects the correlation length between helical twists, includes again a constant prefactor,^44^ and is defined by

5 $$\left\langle \cos\left(\Delta \omega_{i}\right)\right\rangle =\alpha_{tw}\exp\left(-\frac{l_{i0}}{l_{tw}}\right)$$

where ω_*i*_ is the instantaneous global twist (sum of helical twists) between pairs 0 and *i*, Δω_*i*_ = ω_*i*_-ω_*i*0_, and ω_*i*0_ = ⟨ω_*i*_⟩. Again, a linear fit of the logarithm of the left-hand side as a function of *l*_*i*0_ (semilog plot) is used to deduce *l*_tw_ and α_tw_. Since the equilibrium twist is subtracted, the twist p.l. is analogous to the dynamic bending p.l.

To assess the stretching and twisting elasticity, we assume that the deformation energy *E*_rod_ of the duplex is a general quadratic function of the global length *l* and the global twist ω.^44,98^ Introducing the (column) global coordinate vector **w**_rod_=(*l*, ω)^T^, the deformation energy takes the form

6 $$E_{rod}=\frac{1}{2l_{0}}\left(w_{rod}-{ŵ}_{rod}\right)\cdot K_{rod}\left(w_{rod}-{ŵ}_{rod}\right)$$

where ${ŵ}_{rod}={\left(l_{0},\omega_{0}\right)}^{T}$ is the vector of equilibrium configuration, with components

7 $$l_{0}=\left\langle l\right\rangle ,\omega_{0}=\left\langle \omega \right\rangle$$

and **K**_rod_ is the 2 × 2 global stiffness matrix, related to the coordinate covariance matrix **Σ**_rod_ as

8 $$K_{rod}=k_{B}Tl_{0}\Sigma_{rod}^{-1}$$

Equations 6–8 are analogous to eqs 1–3, except that eq 6, and consequently also eq 8, contain the equilibrium length *l*_0_ of the duplex. This ensures that the entries of **K**_rod_ are material constants and not apparent stiffnesses (related to, for example, extending the duplex by a given distance), which would be inversely proportional to the duplex length.

The diagonal entries of **K**_rod_ are stiffness constants with respect to stretching and twisting, respectively, while the other coordinate is constrained (2D deformation). However, experiments on duplex elasticity are usually done in such a way that the undeformed coordinate is relaxed (1D deformation, e.g. torsionally relaxed stretching^111^). For any fixed value of the deformed coordinate, the undeformed one then takes the value that minimizes the elastic energy. Minimizing the energy given by eq 6 and using eq 8, we obtain relations for the 1D stretch modulus *Y*

9 $$Y=\frac{k_{B}Tl_{0}}{var\left(l\right)}$$

where var­(*l*) is the variance, or square standard deviation, of *l*. Thus, *Y* is expressed in units of force. The formula for the 1D twist stiffness *C* is entirely analogous, except that it is convenient to divide it by *k*_B_ *T*, i.e. *C* is expressed in units of length

10 $$C=\frac{l_{0}}{var\left(\omega \right)}$$

This definition brings *C* in close relation to the twist persistence length *l*_tw_. It can be shown (see e.g. refs 112, and 113 ) that, for an intrinsically straight, homogeneous, isotropic elastic rod, the two are related as *C* = *l*_tw_/2.

We see that eqs 9 and 10 are in fact 1D analogies of eq 8, that is, the 1D deformation proceeds as if the deformed coordinate was the only one present, while the other, undeformed coordinate belongs to the thermal bath.

To probe the coupling between twist and stretch deformation (twist-stretch, or TS coupling), the full 2D stiffness matrix **K**_rod_ must be considered. If the excess twist Δω = ω – ω_0_ is constrained, then the elongation Δ*l* = *l* – *l*_0_ takes the value that minimizes the elastic energy (eq 6). Performing the minimization (see ref 108), we find that the twist-stretch coupling coefficient *k* = Δ*l*/Δω is given by the (negative) ratio between the off-diagonal entry and the one related to stretching

11 $$k=-\frac{{\left[K_{rod}\right]}_{12}}{{\left[K_{rod}\right]}_{11}}$$

or equivalently, using eq 8

12 $$k=\frac{{\left[\Sigma_{rod}\right]}_{12}}{{\left[\Sigma_{rod}\right]}_{22}}$$

where the denominator is just the variance of the global twist ω and the numerator is equal to the covariance between ω and *l*. It is convenient to express *k* in nanometers per turn. Thus, if distances are measured in Å and angles in radians, one should multiply *k* obtained from eqs 11 or 12 by π/5.

### Data Validation

A number of studies compared the structure of MD-simulated DNA and RNA oligomers to crystallographic, NMR, or EPR data (e.g., refs 33,54,85–87,90,114, and 115 ). Although the comparison may not be straightforward, the general conclusion is that, among other parametrizations, the DNA and RNA force fields employed here (OL15 for DNA,^80^ χOL3 for RNA^88,89^ ), and the simulation setup used, give a reliable prediction of the duplex structural dynamics. Thus, we will not repeat these extensive validations here. Rather, we already assume that the raw MD data of an NA duplex represent its structure and dynamics rather well, and we test our model predictions mainly against this data. Specifically, we employ independent MD simulations of DNA and RNA oligomers including all pentanucleotide sequences (set52, see above) as the validation set. We do, however, refer to experiment frequently, especially in the application part of the work.

To reliably estimate the model parameters from MD, the mean and stiffness data have to be converged. To check this, we compute a given quantity *x* for the first half and the second half of each trajectory (*x*_1_ and *x*_2_), as well as for the full trajectory (*x*_full_). The absolute error is then estimated as ε_*a*_ = 0.5­(|*x*_1_ – *x*_full_| + |*x*_2_ – *x*_full_|), that is, the mean error between the value for the full (filtered) trajectory and for its halves. The coordinate means are converged with extreme accuracy (<0.008 Å and <0.09°, Table S4). To verify the convergence of the coordinate stiffnesses, we compute the relative error ε_r_ = ε_a_/*x*_full_ and found it to be <1% (Table S5), again a very good convergence. The global stiffness constants converge mostly within 2–4%, with the exception of the TS coupling which exhibits a <5.5% error (Table S6). These errors are higher than those for the (local) coordinate stiffnesses and indicate a relatively slow convergence of these global quantities where not only local, but also global equilibrium must be reached. Nevertheless, the errors are still much smaller than the sequence variability of the global constants, which reaches 50% for the TS coupling and 20% for the others (see below).

The overwhelming majority of hexamers are present only once in our data set, but the few duplicates we have (184 out of 4096) enable us to at least partially check for the consistency of the hexameric scale assumed here. Any deviation between duplicates would indicate effects beyond this scale, be it a lack of convergence (e.g., hexamers in the middle of the duplex could behave differently from those close to the ends) or sequence context effects beyond the hexameric range. The means of the step coordinates for the central step of the hexamer exhibit absolute deviations <0.05 Å and <0.5° between the duplicates (Table S7), the coordinate stiffnesses show relative deviations of <6% (Table S8). These errors can be considered rather low, especially given the uncertainty of many experimental structural or stiffness data.

## Results and Discussion

### Two Length Scales of Effective Base–Base Interactions

In many earlier studies, the range of interactions between rigid bases and base pairs to be included in the proposed model was chosen a priori. Here we start by asking a simple question: what is the effective interaction range between bases in DNA and RNA double helices? To this end, we first analyzed stiffness matrices of a 33 bp oligomer in its DNA and RNA versions from our earlier work^79^ (here denoted sequence s0, Figure 2).

First of all, bases in DNA and RNA double helices are involved in nearest-neighbor, or dimeric, interactions of Watson–Crick hydrogen bonding and intrastrand as well as interstrand stacking (red connecting lines in Figure 2A), which have been studied in great detail.^116–119^ If these were the only interactions present, the entries of the stiffness matrix outside the dimeric range (delineated by red contours in Figure 2B) would be exactly zero, as discussed in ref 63. This is clearly not the case, as there are visibly nonzero entries outside this range.

If the dimeric range is imposed by setting all entries outside the red band to zero, the resulting matrix remains positive definite, but its eigenvalues are far away from those of the full stiffness matrix (Figures S1, and S2). One might think that adding the trimeric interaction range (green lines in Figure 2A,B) could only improve matters. However, the opposite is true—the resulting matrix is not positive definite and therefore cannot represent a valid stiffness matrix. Thus, important longer–range interactions are missed. Adding the tetrameric interaction range (blue in Figure 2A,B) produces a positive definite matrix for RNA, but not for DNA, and the eigenvalues are still quite off (Figures S1, and S2). It is only the pentameric interaction range (magenta in Figure 2A,B) or longer that again produce a safely positive definite matrix both for dsDNA and dsRNA. The gradual improvement of the description as we include longer and longer interaction ranges is clearly seen in the fact that the eigenvalues of the truncated matrices approach those of the full matrix (Figures S1 and S2). Motivated by these observations, we tested all the DNA and RNA duplexes analyzed in this work (set107, set52, set14), with entirely analogous results (Figure 2C).

The most likely contributors to DNA stiffness are base stacking interactions and electrostatic repulsions between charged phosphates, although their exact roles are still debated.^120,121^ Stacking interactions are short-range, but charge repulsion could in principle contribute to long-range couplings. For instance, the DNA and RNA bending persistence length decreases with increasing salt concentration, while the stretch modulus increases,^122,123^ and the DNA twist rigidity appears to be independent of salt.^124^ A useful method to probe the electrostatic effects is a partial or complete neutralization of the phosphate charges.^120,125,126^ The bending persistence length of dsRNA is higher than that of dsDNA, but a recent study reported that this difference decreases with increasing monovalent salt, and the two become identical for simulated oligomers where a +1 charge was uniformly dispersed over the whole phosphate group.^127^

To test the role of electrostatic interactions in long-range base–base elastic couplings, we simulated the s0 sequence in its DNA and RNA forms with the neutral phosphates and no ions present, as well as with neutral phosphates and added 150 mM KCl ions (Methods). The mere neutralization of the nonbridging oxygens we probe here necessarily introduces a disbalance in the charge distribution of the duplex, and a proper approach would include not only a reparametrization of the remaining charges, but also of the backbone torsions. Nevertheless, here we assume that if the electrostatic interactions were dominant, we would see a major effect even using these imperfectly parametrized neutral duplexes. However, we find that the definiteness of the stiffness matrices is the same as for the standard, charged duplex (Figure 2C), and even the eigenvalues take similar values (Figures S1, and S2), indicating only a minor effect of electrostatic interactions on the base–base elastic couplings in DNA and RNA duplexes at physiological salt concentrations.

Together, these observations indicate that at least the pentameric scale, i.e. roughly half the helical turn, is necessary to fully capture the effective base–base elastic couplings in DNA and RNA double helices at physiological conditions, and that these couplings are not dominated by electrostatic interactions.

Our interpretation of these findings is as follows. At the dimeric scale, it is the short-range interactions (stacking and base pairing) that dominate the duplex rigidity. However, their contribution is insufficient to fully explain the duplex mechanical properties. This is seen in the deviation of the dimeric eigenvalues from those of the full stiffness matrix (Figures S1, and S2). As soon as we step out of the dimeric scale, there is another, long-range mechanism that starts to dominate, and it requires at least a tetrameric (RNA) or pentameric (DNA) scale to be captured. Our data suggest that this long-range coupling mechanism is predominantly not of electrostatic origin. It is possible that the couplings stem from the mechanical constraints imposed by the backbone, a phenomenon known from polymer physics^128^ and considered also in DNA modeling.^60^ The longer coupling range for the DNA duplex may be related to the different helix geometry, or to the correlated conformational substates not present in the RNA duplex.^67,68,129^

### Sequence-Averaged DNA and RNA Double Helices

To obtain sequence-averaged mean (or equilibrium) coordinates, we average the mean step coordinates and major groove widths over all hexameric contexts, and intra-basepair coordinates and minor groove widths over all heptameric contexts.

To compute the coordinates for a single structure (e.g., an MD snapshot), one strand of the duplex has to be chosen as a reference. Upon changing the reference strand, some coordinates change sign (the odd ones—buckle, shear, shift, tilt, Y-disp and tip), while the other, even ones remain unchanged. The same applies to trajectory means, i.e. equilibrium coordinates. Thus, averaging over all (not just the unique) hexamers and heptamers implies that the mean odd coordinates are exactly zero. Their standard deviation is then equal to root-mean-square distance from zero.

Table 1 shows the sequence-averaged values, and standard deviations to capture sequence-specific variability, computed over all hexamers or heptamers. The values conform to the B-DNA and A-RNA structures. Here we only briefly comment on rise and twist, parameters that have been measured for free duplexes in solution.

**Table 1:** Sequence-Averaged Structural Parameters of the Simulated DNA and RNA Helices, Computed over All Hexameric or Heptameric Sequences

	DNA	RNA
shear [Å]	0 ± 0.12	0 ± 0.12
stretch [Å]	–0.02 ± 0.04	–0.02 ± 0.05
stagger [Å]	0.03 ± 0.10	–0.07 ± 0.10
buckle [°]	0 ± 5.86	0 ± 4.79
propeller [°]	–11.03 ± 3.70	–12.61 ± 1.75
opening [°]	0.24 ± 0.84	0.28 ± 0.83
shift [Å]	0 ± 0.33	0 ± 0.09
slide [Å]	–0.13 ± 0.36	–1.64 ± 0.14
rise [Å]	3.33 ± 0.10	3.30 ± 0.10
tilt [°]	0 ± 1.61	0 ± 0.72
roll [°]	3.14 ± 2.53	8.83 ± 2.95
twist [°]	34.49 ± 2.84	30.08 ± 1.01
X-disp [Å]	–0.84 ± 0.53	–4.42 ± 0.31
Y-disp [Å]	0 ± 0.41	0 ± 0.17
h-rise [Å]	3.24 ± 0.11	2.67 ± 0.14
inclination [°]	5.72 ± 4.52	15.93 ± 4.63
tip [°]	0 ± 2.62	0 ± 1.34
h-twist [°]	35.54 ± 2.75	32.24 ± 1.64
minor g. width [Å]	12.04 ± 0.89	17.11 ± 0.22
major g. width [Å]	18.36 ± 0.61	18.11 ± 0.31

The DNA helical rise (h-rise, Table 1), or distance between base-pair centers projected onto the helical axis, agrees quantitatively with recent measurements of base-pair distance using gold labels and anomalous small-angle X-ray scattering (3.23 ± 0.1 Å),^101^ while the RNA h-rise is somewhat underestimated compared to the consensus experimental value of 2.8 ± 0.1 Å.^103^ The local rise, capturing the distance between successive base pairs along their mean normal, primarily reflects the local stacking and is very similar in the DNA and RNA helices.

Twist is an interesting case. The MD-derived helical twist (h-twist, Table 1), or the twist angle about the helical axis, implies 11.2 bp/turn for RNA, in agreement with the experimental value of 11.3 ± 0.1 bp/turn.^102^ In the case of DNA, however, it is the local twist that yields 10.44 bp/turn, consistent with 10.5 bp/turn predicted for a random sequence with 50% GC content based on DNA cyclization,^5^ and in agreement with 10.4 ± 0.1 bp/turn from gel electrophoresis^10,99^ and 10.6 ± 0.1 bp/turn from enzyme digestion.^100^ The DNA helical twist, in contrast, is ∼1° higher and gives just 10.13 bp/turn, although the two types of twist are generally assumed to be similar for B-DNA. This issue may deserve further investigation.

The sequence-dependent variability of the step coordinates (in absolute terms) is in general smaller for RNA than for DNA, with a notable exception of roll, as already found at the tetrameric level.^129^ Our data indicate smaller RNA structural variability also for intra-basepair and helical coordinates, except inclination (Table 1).

### Sequence-Dependent Structure and Flexibility

The complete hexanucleotide coverage allows us to examine the sequence dependence at an unprecedented context range with minimal model assumptions. We first present equilibrium coordinates for individual dinucleotide steps. Each value is the average over all the hexamers with the given step in the middle (Table 2). Recently published crystallographic data from the Olson lab^130^ are also shown. The two data sets exhibit common trends, and the X-ray and MD averages (last lines of Table 2) agree within the intervals of dinucleotide variability. Analogous data for RNA (MD only) are in Table S9.

**Table 2:** Dinucleotide Equilibrium Conformations Deduced from a Crystal Structure Ensemble (Ref 130) and from MD (This Work)^a^

middle dimer	source	shift [Å]	slide [Å]	rise [Å]	tilt [°]	roll [°]	twist [°]
CG	Olson	0	0.36	3.34	0	6.4	34.3
	MD	0	0.24 ± 0.12	3.33 ± 0.12	0	6.16 ± 1.04	34.31 ± 3.11
CA	Olson	–0.06	0.18	3.33	0.0	5.6	35.1
	MD	–0.23 ± 0.20	0.15 ± 0.17	3.29 ± 0.12	–0.11 ± 1.13	6.70 ± 0.98	33.13 ± 3.28
TA	Olson	0	0.20	3.35	0	2.3	37.5
	MD	0	0.29 ± 0.17	3.25 ± 0.10	0	5.26 ± 0.87	34.51 ± 2.46
AG	Olson	–0.03	–0.34	3.30	–0.4	3.6	32.2
	MD	–0.32 ± 0.19	–0.37 ± 0.21	3.37 ± 0.09	–2.70 ± 0.62	3.93 ± 1.09	33.46 ± 1.83
GG	Olson	–0.01	–0.33	3.36	0.0	4.9	33.2
	MD	–0.20 ± 0.24	–0.25 ± 0.31	3.45 ± 0.10	–0.06 ± 1.08	4.85 ± 0.76	34.90 ± 1.65
AA	Olson	0.02	–0.27	3.24	0.0	–0.2	35.1
	MD	–0.36 ± 0.19	–0.06 ± 0.16	3.31 ± 0.05	–2.27 ± 0.54	1.63 ± 0.80	35.99 ± 1.44
GA	Olson	–0.02	–0.11	3.28	–0.1	1.9	36.3
	MD	–0.45 ± 0.17	0.23 ± 0.17	3.32 ± 0.06	–1.08 ± 0.95	2.22 ± 0.75	37.74 ± 1.81
AT	Olson	0	–0.69	3.22	0	0.1	30.7
	MD	0	–0.71 ± 0.06	3.24 ± 0.05	0	–0.53 ± 0.71	30.70 ± 1.36
AC	Olson	0.00	–0.64	3.26	0.2	1.7	31.9
	MD	0.19 ± 0.17	–0.49 ± 0.13	3.29 ± 0.08	–0.40 ± 0.77	0.22 ± 0.58	32.69 ± 1.88
GC	Olson	0	–0.45	3.29	0	2.7	33.3
	MD	0	–0.28 ± 0.17	3.35 ± 0.08	0	0.20 ± 0.39	36.60 ± 1.80
Avg	Olson	–0.01 ± 0.02	–0.21 ± 0.36	3.30 ± 0.05	–0.03 ± 0.15	2.90 ± 2.22	33.96 ± 2.10
	MD	–0.14 ± 0.20	–0.13 ± 0.35	3.32 ± 0.06	–0.66 ± 1.02	3.06 ± 2.66	34.40 ± 2.05

a The MD values are means and standard deviations over all the hexamers with the indicated step in the middle. The last line shows the average and std over the 10 dinucleotide means.

Next, we turn to the tetrameric level. We present the values for the central step within a given tetramer, and the central pair within a given pentamer, each averaged over all the hexameric (heptameric) contexts. We then separately present the hexameric/heptameric variability in terms of the difference between the highest and the lowest value for the given tetramer/pentamer. As for the coordinate flexibility, we quantify the context dependence in relative terms, i.e. maximal minus minimal value divided by the average. Figure 3 illustrates the information we provide, analogous plots for the remaining coordinates are at https://zenodo.org/records/15575888. We find this type of visualization (a “heatmap”) useful, since it enables the reader to discern sequence-dependent patterns at a glance. Taking twist as an example (Figure 3), we see the domain of low (the YYRR tetramers, upper left corner of Figure 3A) and high DNA twist (RYRY, lower left corner). In some cases, the values are largely dictated by the central step (DNA AT and many RNA steps, Figure 3A,B), while a clear tetramer dependence is observed in other instances. The DNA twist stiffness (Figure 3C) can differ by a factor of 5 between tetramers and is exceptionally high for the AATT tetramer, a short symmetric A-tract.

In contrast to earlier tetramer-oriented studies,^54,129^ our data enable us to also probe hexameric/heptameric context effects for a given tetramer/pentamer. We find that, although the RNA hexameric/heptameric structural variability is in general smaller than for DNA, it is certainly not negligible. For instance, RNA buckle can vary by 6°, propeller by 3°, roll and tilt by 2°, depending on the hexameric/heptameric context. The variation of the DNA twist stiffness for a given central tetramer can reach up to 45%, is on average highest for YYRR (Figure 3D, upper left corner), and lowest for RRYY (Figure 3D, lower right corner). The RNA hexameric stiffness variability is roughly half the DNA one for all the step coordinates, but can still reach 35% in the case of shift and ∼20% for roll and twist. We also point out the extreme flexibility of DNA and RNA buckle (<0.01 kcal/mol/deg^2^).

The relevance of the hexanucleotide data compared to the tetramer level is further illustrated in Figure 4, showing the hexamer context dependence of slide, roll, and twist mean (or equilibrium) values and stiffnesses for selected tetramers. Notice that all the tetramers in one panel of Figure 4 shear the same central step. The Figure exposes values for all individual hexameric contexts of the indicated tetramer. To make the data directly comparable among tetramers, the average over the hexameric contexts of the given tetramer has been subtracted. It is seen that, while some tetramers are nearly unaffected by the hexameric context (e.g., when it comes to mean twist, the TCAT tetramer), others are strongly affected: For example, the twist of the CA step within the CCAC tetramer may still differ by 5.5° depending on the hexameric context. Similarly, the mean roll of AG within CAGC may differ by 4° depending on the hexameric context, while the roll of AG within AAGA differs by no more than 1°. An analogous plot for RNA (Figure S3) indicates a weaker hexameric context dependence than for DNA.

The sequence-specific minor groove widths are in Figure 5. While the DNA minor groove is mostly determined by the outer bases of the pentamer (Y···R > R···R > R···Y on average), the RNA values are largely dictated by the central trimer, or even by the central pair (A-U or G-C). Nevertheless, the heptameric variation (for a fixed central pentamer) can still reach 1.2 Å for DNA and 0.5 Å for RNA, and the heptameric variability in minor groove stiffness is quite substantial, namely 60% and 30% for DNA and RNA, respectively (https://zenodo.org/records/15575888). As for the major groove, its width and stiffness depend on the whole hexameric sequence and we only present their histograms (Figures S4, and S5). Tables in the csv format presenting means and stiffnesses of intra- and inter-basepair coordinates and groove widths for all the individual hexamers or heptamers are at https://zenodo.org/records/15575888.

### Global Elasticity

#### Sequence-Specific Variability of Material Constants

At the global level, we characterize the simulated 107 DNA and 107 RNA duplexes in terms of their bending and twisting persistence lengths, stretch modulus, twist stiffness, and the coupling between twisting and stretching (TS coupling, Methods). The large, balanced set of sequences containing all hexamers, most of them exactly once, enables us to probe sequence-dependent variability of these constants, significantly extending the available information.

To infer the stretch and twist moduli and the TS coupling, we examine thermal fluctuations of the total length and twist of the oligomer, as in ref 98. These global coordinates have to be carefully chosen. Here we define the oligomer length as the sum of helical rises, and its global twist as the sum of helical twists (in radians), using the 3DNA definitions.^48^ This choice, proposed in ref 108, gives the correct magnitude and sign of the TS couplings for DNA and RNA,^108^ and realistic values of stretch and twist moduli for DNA as well as RNA and hybrid duplexes.^44,79^

Table 3 summarizes the mean values and standard deviations of the material constants for the set of 107 DNA and 107 RNA sequences, their distributions are in Figures S6. For each oligomer, the central 25 bp part was examined, the GCGC caps were excluded. The filtered trajectory (Methods) was used in each case.

**Table 3:** Global Material Constants for the Set of DNA and RNA Oligomers Containing all Hexanucleotides Simulated in This Work (Regular Font)^b^

parameter	symbol	DNA	RNA
stretch modulus (pN)	*Y*	1621 ± 127	694 ± 37
		*900–1500*	*350–500*
twist stiffness (nm)	*C*	119 ± 7	118 ± 9
		*94–109*	*100 ± 2*
half twist persistence length (nm)	*l*_tw_/2	136 ± 8	122 ± 11
twist-stretch coupling (nm/turn)	*k*	0.81 ± 0.10	–1.11 ± 0.20
		*0.42 – 0.5*	*–0.85 ± 0.4*
dynamic bending persistence length (nm)	*l*_d_	68 ± 4	84 ± 4
		*46 – 54*, *78 ± 13*	*54 – 64* ^a^

a Total persistence length.

b Means and standard deviations for the 107 DNA and 107 RNA sequences are shown. Experimental values compiled from the literature (see text) are included for comparison (italics).

The sequence-averaged values reproduce well-known experimental findings:^5,6,131,132^ RNA is much more flexible in stretching than DNA, has a similar twist modulus, and its TS coupling is of similar magnitude and opposite sign. The values are somewhat overestimated compared to the experimental range, as already reported for a limited set of DNA and RNA sequences.^44,79,133–135^ However, here we also provide the sequence-specific variation over a set of oligomers containing all hexanucleotides. Taking three standard deviations as the variability measure, we obtain a spread of roughly 20% for *Y* and *C*, while the TS couplings may vary by as much as 50%.

The dynamic bending persistence length (p.l.) *l*_d_ captures the decay length of directional correlations due to thermal fluctuations. Together with the static disorder quantified by the static p.l. *l*_s_, they make up the total p.l. *l*_p_ through the relation 1/*l*_p_ = 1/*l*_d_ + 1/*l*_s_.^136^ Taking the DNA value of *l*_d_ = 78 nm from cryo-EM experiments,^132^ and the consensus value *l*_p_ = 50 nm, implies *l*_s_ = 139 nm. In contrast, cyclization experiments indicate a very large *l*_s_ (>1000 nm), so that *l*_p_ = *l*_d_ with high precision,^137^ a discrepancy probably due to different experimental conditions.^138^ The large *l*_s_ is also supported by an earlier simulation study.^109^ If so, then the 46–54 nm experimental sequence-dependent range (taken from ref 5) corresponds to *l*_d_ and the MD value of *l*_d_ is again somewhat overestimated. Its sequence-dependent variability of 18% found here (histograms in Figure S7) is consistent with the earlier computations^109^ and comparable to the experimental one.^5^

The experimental total persistence length for RNA (Table 3) is higher than the DNA one in physiological conditions.^103,127,131^ We are unaware of any experimental value of RNA dynamic p.l., but our computed value is again higher than the DNA one, as in earlier simulation studies.^79,104^ Furthermore, the sequence-dependent variation we report here indicates a similar spread of values as for the DNA dynamic p.l.

We stress that the dynamic bending persistence length definition employed here (Methods, eq 4) is valid for duplexes of any length, as long as the semilog plot exhibits a linear decay, which is indeed the case (Figure S8). The factorization of the static structure improves the linear fit for DNA, and is absolutely crucial for RNA, since otherwise the decay would be overshadowed by the high-amplitude periodic signal related to the A-RNA geometry (Figure S8). If the molecule is intrinsically straight ( ${\mathrm{t̂}}_{i}\cdot {\mathrm{t̂}}_{0}=1$ in eq 4), then the dynamic and the total persistence lengths coincide, as in the case of the worm-like chain model. The twist persistence length *l*_tw_ we deduce here is also well-defined, as indicated by the good linear fit in the semilog plot (Figure S9).

The twist stiffness *C* and twist persistence length *l*_tw_ are often used interchangeably but are two different quantities, the former capturing the twist mechanical rigidity and the latter the decorrelation length of twist fluctuations. They are closely related for an intrinsically straight, homogeneous, isotropic elastic rod, where *C* = *l*_tw_/2 (derived e.g. in refs 112, and 113 ). Here we find that for RNA the two indeed nearly coincide, but our *l*_tw_/2 is distinctly higher than *C* for DNA (Table 3). Since the average twist is subtracted in *l*_tw_ computation (eq 5 in Methods), the twist p.l. is analogous to the dynamic bending p.l. We are unaware of any experiments measuring the twist persistence length directly, without reference to twist stiffness.

#### Static Disorder

The hexanucleotide sequence coverage also enables a robust analysis of the static disorder due to sequence-specific variability of the equilibrium, or static structure of the duplexes, which we characterize by the static bending and twisting persistence lengths (Supporting Information Results). To this end we generated 10^5^ random sequences, each 500 bp long, and considered each of them as a snapshot from a virtual MD simulation. We then processed these snapshots exactly as we did for the MD-generated ones to compute the bending and twisting persistence lengths (eqs 4 and 5 in Methods). The structure that is factorized out is now the sequence-averaged static structure. This enables us to get rid of the wiggles, pronounced especially for RNA, and obtain a clean linear decay in the semilog plot (Figure S10). We find the static bending persistence length very high (around 900 nm both for DNA and RNA, Supporting Information Results). Thus, the bending disorder is very weak, in line with the scenario proposed for DNA in.^137^ The twisting disorder which, we believe, has not been examined before, is even weaker (even higher static twist persistence length, Supporting Information Results). Thus, the MD-derived dynamic bending and twist persistence lengths in Table 3 should also be understood as total persistence lengths predicted by the MD data.

#### Length-Scale Dependence

Early MD simulations already indicated that DNA elastic properties are length-dependent: short fragments of a given oligomer are more flexible, the rigidity increases with the fragment length, and it only gets saturated at the scale of half the helical turn or more.^98^ The phenomenon was later rediscovered using much longer oligomers and simulated time scales,^139^ and was a subject of intense theoretical study.^58–61^ Still, many questions remain open. Most studies focus on bending and twisting rather than stretching. The length-dependence of TS coupling, to our knowledge, has not been examined. Moreover, a limited set of sequences have so far been probed.

Here we study the length-dependent elasticity of DNA and RNA duplexes on our sequence set covering all hexanucleotides. We find that the bending and twisting persistence lengths are nearly length-scale independent, as we do not observe any obvious change of slope in our semilog plots used to infer them (Figures S8 and S9).

To probe the length dependence of the stretch modulus *Y*, twist stiffness *C*, and TS coupling *k*, we proceed much the same way as in the early work.^98^ For a given simulated sequence, we compute *Y*, *C*, and *k* for all its subsequences (fragments), starting from dinucleotides and up to the full sequence length. The values for fragments of the same length are then averaged. In this way, we obtain length-dependent profiles of *Y*, *C*, and *k* for the given simulated duplex (Figure 6, gray lines). We repeat the procedure for all the 107 DNA and 107 RNA duplexes, and the values are finally averaged to obtain sequence-independent profiles (red lines in Figure 6).

We find that the stretch modulus, twist stiffness, and TS coupling depend significantly on the fragment length. Both the stretch modulus and the twist stiffness increase with length, that is, DNA and RNA duplexes are more deformable in stretching and twisting at short scales than at long scales. Importantly, this short-scale softening is not due to broken pairs or noncanonical structures, since these have already been filtered out from the data. Rather, these are properties of intact DNA and RNA double helices. The increase in stiffness ranges from some 30% for RNA stretch modulus up to a 3-fold increase for DNA twist stiffness (Figure 6). The TS coupling slowly decreases for longer fragments, but a nonmonotonic behavior is observed at shorter scales. Although the values of the elastic constants are sequence-dependent, their length dependence appears to be universal. The data in Figure 6 also suggest that the 25 bp scale is about the minimal length to obtain saturated (or bulk) values.

### A Predictive Model of Sequence-Dependent Shape and Stiffness

#### Shape

We estimate the static (equilibrium) structure, or shape, of an arbitrary sequence from the hexanucleotide MD data using a hexameric sliding window for base-pair step coordinates and major groove widths, and a heptameric sliding window for intra-basepair coordinates and minor groove widths. The heptameric data are computed as averages over the two overlapping hexamers forming the heptamer (Figure 1B). The stiffness associated with the individual coordinates (Methods) is estimated analogously.

Previous studies used sliding windows of tetrameric (e.g., refs 54, and 67 ) or pentameric length.^22,68^ The obvious limitation of a sliding window is that no context effects beyond the window length can be captured. At least two methods have been proposed to overcome this constraint (Introduction). One of them is based on the inverse of a banded, but not block diagonal matrix,^64,65,104^ the other one employs a deep learning approach.^22^ Both are complex models involving highly nontrivial parameter optimization. Here we are limited by the window size, but our sliding windows are longer than any used before, the approach is entirely straightforward and involves no model assumptions other than the underlying all-atom MD data (and the heptamer approximation).

To test the predictive power of our model, we produced a validation set of independent atomistic MD simulations of 52 DNA and 52 RNA oligomers containing all pentameric sequences (set52, Methods). For each sequence from set52, we predicted its shape parameters from the hexameric/heptameric model and compared to the actual values from MD. The results are in Tables S10 and S11. The average error in DNA step coordinates is <0.5° and <0.05 Å, the RNA values are even smaller. The intra-basepair coordinates are also well predicted. The sequence-dependent DNA minor groove width is captured within 0.16 Å on average, the major groove within 0.26 Å, the RNA values are again even better.

#### Stiffness

Just as for the shape parameters, we tested the prediction of the stiffness constants associated with individual coordinates (Methods). The relative errors ε = |*k*_pred_ – *k*_set52_|/*k*_set52_ are listed in Table S12. The DNA step stiffnesses are predicted within 3.5% (twist 1.2%), the intra-basepair ones within 5%, the groove stiffnesses within 1.5%. The RNA data are again considerably better. Interestingly, the predictive errors on shape and coordinate stiffness are similar to the variations between duplicate hexamers (Methods). Thus, the predictive errors are already comparable to the intrinsic uncertainty of the model itself.

Encouraged by these results, we moved on to predict not only the shape and coordinate flexibility, but the whole, nonlocal stiffness matrix of an arbitrary sequence. To this end, we took the hexameric stiffness blocks and assembled them together, arithmetically averaging the overlapping parts (Figure 1A, Methods). We again used the set of 52 DNA and 52 RNA oligomers (set52) covering all pentamers to validate the model. To our disappointment, the smallest eigenvalues of some of the assembled matrices (nondimensionalized, Methods) for the set52 sequences were very close to zero, or even negative (Figure S11). At the same time, the eigenvectors were reproduced rather well (Figure S12). The problematic eigenmodes mostly involved the very flexible intra-basepair coordinate buckle, presumably less relevant in many applications.

To deal with the problem, we decided, after some experiments, to introduce a cutoff on the eigenvalues. We factorized the assembled stiffness matrix **K̃** as **K̃** = **PD̃P** ^T^, where **D̃** is the diagonal matrix containing the eigenvalues and **P** is an orthogonal matrix whose columns are the corresponding unit eigenvectors (spectral decomposition). Now the eigenvalues in **D̃** smaller than the cutoff λ_c_ were replaced by λ_c_, obtaining a new diagonal matrix **D̃**, and the new stiffness matrix was computed as $\mathrm{K̃}=P\mathrm{D̃}P^{T}$ . An analogous method was earlier proposed to construct a nearest positive semidefinite matrix to a given symmetric matrix, where negative eigenvalues were replaced by zeros.^140^ Thus, we believe that this method yields a stiffness matrix with the desired properties by introducing a minimal perturbation to the original one.

When the cutoff is applied in this way, the resulting stiffness matrix is in general not banded any more. However, we checked that setting the entries outside the hexameric band to zero introduces a small change both in eigenvalues and eigenvectors, and can be safely made should one need a more compact representation of the matrix data. Interestingly, we found that this trimming in fact even slightly improves the agreement with the validation set, suggesting that the entries outside the hexameric band are mostly noise.

To optimize the cutoff, we made use of the set14 sequence set containing all unique tetramers. We chose the cutoff so that the Pearson correlation coefficients between the global material constants (bending persistence length, stretch modulus, twist stiffness, etc.) for the set14 sequences computed from the model and those inferred directly from MD be maximal (Supporting Information Methods). To obtain the model values, we generated the multidimensional Gaussian distribution of intra-basepair and step coordinates, using the shape parameters and stiffness matrix predicted by the model, then deduced the material constants from this structural ensemble. The optimization is numerically rather insensitive, yielding λ_c_ = 0.48 for DNA and λ_c_ = 0.44 for RNA as good choices (Figures S13, and S14).

With these cutoff values, the correlation coefficients of the predicted material constants for the validation set52, and the values deduced directly form MD of the same set, are high, 0.8–0.9 in most cases (Figures S15, and S16). Furthermore, the mean relative error on the eigenvalues themselves are 4.1% for DNA and 2.9% for RNA. This indicates that the model can reproduce both local and global flexibility of DNA and RNA duplexes rather well.

#### Applications of the Model

We have developed a model of sequence-dependent structure and harmonic deformability of DNA and RNA duplexes and parametrized it from all-atom, explicit-solvent MD simulation data of 107 DNA and 107 RNA oligomers containing all hexanucleotide sequences. Here we present some applications of the model to demonstrate its utility.

#### The Dickerson Dodecamer

This iconic DNA oligomer was the first one to be crystallized^2^ and serves as a touchstone for any model of sequence-dependent DNA structure (e.g., refs 114, and 141 ). A number of crystal structures, as well as NMR data, are available. Here we compare our model prediction with high-resolution crystal structures, selected as in ref 114 to cover different space groups,^142–145^ together with high-quality NMR data.^4^ The model predictions are consistent with these experimental structures (Figures S17, and S18). The model performs particularly well compared to the NMR solution data for the coordinates roll, twist and slide, decisive for DNA structural adaptation in complexes with protein.^95^

#### Signature Triple Minimum of Minor Groove Profile at the Exd–Scr Binding Site

Transcription factors may recognize their target DNA sequences via specific, sequence-dependent shape or deformability features (shape readout^13^). Of these, the *Drosophila* Hox protein Sex combs reduced (Scr) has distinct DNA recognition properties when it binds as a heterodimer with its cofactor Extradenticle (Exd).^71^ The crystal structure of the Exd–Scr complex bound to DNA (2R5Z^146^) suggests three locations of insertion from Exd and Scr arginine residues into the minor groove. The deep-learning based Deep DNAshape tool^22^ predicted minor groove widths preformed (narrowed) at these three sites, but a direct comparison with the crystal data is lacking.

Here we examined the top ten of the highest-affinity sequences for the Exd–Scr complex with DNA reported in ref 71. Although the individual sequences give a somewhat noisy picture, their average clearly shows the signature triple minor groove minimum of the target DNA sequence (Figure 7). Furthermore, the minor groove profile is entirely consistent with the crystal data, although the minima in the crystal are somewhat deeper, as expected, since the protein is already present. Thus, our heptamer-based sliding window approach, parametrized from MD data in a straightforward manner, can quantitatively reproduce this subtle effect.

#### Nucleosome Destabilization by DNA polyA Sequences

Nucleosomes are destabilized by polyA DNA tracts, with profound implications on genome function and chromatin organization.^147–149^ Our group reported that the deformation energy of A-tracts in the nucleosome is position-dependent and is higher on average than that of a control sequence without A-tracts.^150^ However, the nucleosome destabilization is known to be polyA length-dependent and is more pronounced for longer tracts, such as A_34_.^151^

Here we designed a 401 bp random sequence (control, without A-tracts), then mutated its central part to A_*n*_ (*n* = 21, 41, 61). We used our model (hexameric/heptameric sliding window for the structure, stiffness matrix assembly from hexanucleotide blocks, cutoff applied) to predict the shape and stiffness of the sequences. We then threaded the sequences through a nucleosome structure (1kx5 ^152^) and computed the deformation energy as in eq 1. We only considered the step coordinates roll, twist and slide, since these are highly conserved among nucleosome structures.^153^ All the other coordinates were left free to adopt energetically optimal values (partially relaxed model^150^). The nucleosome destabilization by the A_n_ sequences is clearly visible (Figure S19). Averaging the energetic cost over the positions where the whole A_*n*_ tract is within the nucleosome, we find the energy difference Δ*E* proportional to the tract length and close to 1 kcal/mol per base pair (Table S13). Thus, our model not only correctly predicts nucleosome destabilization by the polyA DNA sequences, but also provides an estimate of the associated energetic cost.

#### Contrasting Bending Mechanisms of DNA A-Tracts and RNA AU-Tracts

It has long been known that A-tracts induce a bend to the DNA double helix toward the minor groove at the tract center, although the exact magnitude of the bend and its dependence on the ionic environment are still debated.^6,148,154–157^ Structural hallmarks of the DNA A-tracts are a narrow minor groove and a high negative propeller twist. A recent study reported global bending also for the RNA double helix, this time caused by the (AU)_*n*_ sequences (AU-tracts): atomic force microscopy imaging showed that phased AU-tracts induce a macroscopic curvature, while all-atom MD simulations identified a localized compression of the dsRNA major groove and a large negative propeller twist at the tracts.^12^ These data suggest similarities as well as differences between the two bending phenomena.

To shed more light on the issue, here we constructed a 28 bp random G/C sequence in its DNA and RNA forms, and mutated its center to A_8_ for DNA and (AU)_4_ for RNA, respectively. The key predictions are in Figure 8. The colored bands are values within one standard deviation from the mean over all hexameric (for the roll) or heptameric sequences. This allows one to quantify the notion of extreme values, meaning values outside this interval. The A-tract is predicted essentially straight (small roll), in line with many earlier reports.^148^ By contrast, the AU-tract exhibits very high positive roll in UA steps accompanied by smaller, but still positive roll in AU steps (Figure 8A). Our model further predicts the well-known narrow minor groove in the DNA A-tract,^148^ but also an expanded minor groove in the RNA AU-tract (Figure 8B). The model reproduces a compressed major groove of the AU-tract (Figure S20), and a high negative propeller in both tracts (Figure 8C), as already reported.^12^ However, here we also examine the propeller stiffness and find a striking difference: the A-tract propeller is exceptionally stiff (high force constant), while the AU-tract propeller is very flexible (low force constant, Figure 8D). Finally, we notice that the A-tract structure gradually builds up in the 5′–3′ direction and is saturated only at the fourth adenine, the minimal A-tract length,^148^ a cooperativity not present in the RNA AU-tract (Figure 8B–D).

Taken together, these data suggest contrasting bending mechanisms for DNA A-tracts and RNA AU-tracts. Our model predicts the A-tract as a straight, cooperative unit, exhibiting narrow minor groove and a high negative, stiff propeller twist, the helix bending toward the minor groove being localized mostly outside the tract or at the junctions, in line with experimental studies.^148^ By contrast, we find the AU-tract to be flexible in propeller, noncooperative and itself highly bent, this time toward the major groove it its center. The bending is due to the high positive roll in UA steps, further supported by smaller but still positive roll in AU steps and accompanied by the expanded minor groove. The opposite bending directions are clearly visible in the atomistic structures of the two molecules (Figure 8E,F). Of note, an analogous bending mechanism of a DNA AT-tract seems not possible due to a much smaller positive roll in TA steps, further neutralized by the negative roll in AT steps (Figure S21).

#### Structural Dynamics

The amount of the MD data (107 DNA oligomers, each simulated for 2 μs, and 107 RNA oligomers, 1 μs each) enabled us to detect rare events, such as base-pair opening or alternative (noncanonical) structures. We focused on the central 25 bp parts containing all hexanucleotide sequences.

#### Base Pairing

The base pairs occasionally open for a short time, then close again. We consider a pair open if at least one of the Watson–Crick hydrogen bonds is broken, by which we mean that the distance between the heavy atoms is greater than 4 Å (Methods). A proper representation of hydrogen bonding is critical especially for folding noncanonical RNA structures and is still a focus of intense research.^78,158,159^ Furthermore, the base-pair opening in the duplex is also related to stacking interactions, which seem to be overestimated in current force fields (see for example ref 160). In the duplexes studied here, the population of broken A-T/A-U and G-C pairs is very small: 0.10 and 0.15% respectively for DNA, 0.38 and 0.63% for RNA. Thus, the RNA pairs in duplexes appear less stable during MD than the DNA ones. For the pair to be detected as broken, a base need not flip out completely. A smaller perturbation is enough, similar to the imino proton exchange measurements where the opening angle as small as ±30° is sufficient for the exchange to take place.^161^

We first examine the open-pair lifetimes. Rather than blindly averaging the timespans of all opening events, we adopted (and simplified) a method proposed in ref 162. The opening times are considered as independent, identically distributed random variables. This is in line with the experimental finding that base pairs open one at a time, but neglects the dependence on base-pair identity (A-T/A-U vs G-C) and on sequence context.^163^ We constructed the survival function *S*(*t*) which represents the probability that the pair remains open longer than time *t*. If *S*(*t*) = exp­(−λ*t*), then the mean opening time is τ_open_ = −1/λ.^162^ We computed *S*(*t*) over all opening events, separately for DNA and RNA duplexes, and plotted the logarithm of *S*(*t*). We implemented the pseudocode from ref 162 for efficient computation of *S*(*t*). For short times, ln *S*(*t*) is visibly nonlinear, indicating brief transient escapes, and it is not well-defined for long times (Figure S22). In between, however, there is a time interval where it is close to linear, and we just fitted a straight line there, whose slope λ then defines the mean opening time. We found τ_open_ of 2.3 ns for DNA and 5.3 ns for RNA. Thus, our MD data predict open-pair lifetimes in DNA and RNA duplexes in the nanosecond range, in agreement with imino proton exchange measurements,^163^ further corroborating the validity of the MD simulation methodology used.

To get more insight into the base pairing dynamics, we plotted histograms of the opening times (Figures S23 and S24). The overwhelming majority of the opening events are brief escapes taking one or several snapshots, where only one hydrogen bond (HB) in the pair is broken, i.e. its length exceeds the 4 Å cutoff. We interpret these as mere thermal fluctuations of the paired structure, with no real bp opening. Interestingly, any of the HB in the pair can be broken this way, which explains the A-T/A-U vs G-C broken pair populations (see above) following the 2:3 ratio, i.e. the number of HB in the pair.

There is then a continuum of nanosecond opening events characterized by the mean opening time computed above. On still longer time scales, the A-T/A-U and G-C pairs start to behave differently (Figures S24 and S25). While for G-C we observe an isolated secondary peak of opening times at 6 ns (DNA) and 4 ns (RNA), the A-T/A-U pairs exhibit a continuum of opening times of diminishing frequency up to 20 ns (A-T) and 120 ns (A-U).

We finally investigated the very long opening events of A-U pairs in more detail. They consist in shearing the pair so that the Watson–Crick pairing is lost and a new HB between AN6 and UO2 is formed (Figure S26), as already reported.^79^ The resulting distribution of the intra-basepair coordinate shear thus exhibits a secondary peak around −4.5 Å, far away from the main one around zero (Figure S27), which would entirely skew the coordinate distribution and therefore also the nonlocal stiffness matrix. To avoid this, we filter out snapshots with any HB broken anywhere within the duplex, except the two outer G-C pairs at the ends (Methods). It is possible that these rare opening events will eventually become negligible in a hypothetical very long MD simulation. Filtering them out would then mimic this limiting case.

#### Noncanonical Structures

We scanned the time series of the intra-basepair and step coordinates to find time intervals of anomalous values, indicative of a noncanonical structure (Methods). In all the DNA MD data, we found only four such structures with lifetimes longer than 10 ns (Figure S28). One of them showed interstrand stacking of the pairs, three others were ladder-like structures associated with long-living concerted flips of the backbone torsions α/γ from *g*–/*g* + to *g+/t*, accompanied by the shift of β from *t* toward lower *g*– (around 240°).

Interestingly, no such structures were observed in the RNA duplexes. Instead, a spectacular flip of a sugar pucker into the B-DNA domain, lasting for 250 ns, was seen, associated with a flip of the torsion angle κ involving the 2′-OH group (H2′–C2′–O2′–HO2′) from wildly fluctuating around 50° to locked around 130° (Figure 9). Changes in the propeller of the pair in question, as well as twists in the two steps involved, were also observed (Figure S29). Several other, shorter flips of this kind (<50 ns) were detected. This type of RNA sugar-pucker and κ flip was recently found in the *E. coli* sarcin-ricin loop using cryo-neutron crystallography,^164^ and in other structures using earlier MD simulations,^165^ but the residues involved were all extra-helical. Our MD data suggest that such flips may take place within the RNA duplex as well.

Finally, we stress that no kinks (long-living, sharply bent structures) or bubbles (long-living stretches of at least two consecutive broken pairs) were observed in any of the DNA or RNA duplexes studied here.

## Conclusions

It has long been recognized that the structure and deformability of the DNA double helix is modulated by its base sequence, with fundamental ramifications for DNA biology and nanostructure design. Despite intense research, this dependence has remained incompletely understood. Sequence-specific variations of shape and stiffness have been emerging also for double-stranded RNA, a prominent structural motif.

In this work we performed a set of atomic-resolution, explicit-solvent MD simulations of double-stranded DNA and RNA oligomers containing all the 2080 unique hexanucleotide sequences, and analyzed them in terms of sequence-specific shape and harmonic deformability. We identified two scales of effective base–base interactions within the DNA and RNA duplexes, and exposed fundamental differences in the rules governing DNA and RNA sequence-dependent structure and stiffness. We constructed a model to predict DNA and RNA shape and harmonic stiffness for arbitrary sequence, validated it on an independent data set of MD-simulated oligomers involving all DNA and RNA pentameric sequences, and demonstrated its utility in various experiment-oriented applications related to the duplex structure, stiffness and protein binding. The sheer amount of the simulated data enabled us to also detect and quantify rare events, such as base-pair opening or concerted sugar-pucker and 2′-OH flips in RNA duplexes.

Our results indicate that hexamers may be the minimal independent unit at the rigid base level, in the sense that there is a very small variability in shape and stiffness of the central base-pair step between duplicate hexamers. At the global level, however, some of the material constants, notably DNA twist stiffness, need as many as 25 bp to reach the bulk value.

In contrast to existing complex models involving highly nontrivial parameter optimization, our approach is entirely straightforward and includes just one, rather insensitive adjustable parameter, the stiffness matrix eigenvalue cutoff. We believe that the cutoff may remain unchanged if NA duplexes with gently modified bases, such as 5-methylcytosine or 8-oxoguanine, are investigated in the future. We finally remark that, thanks to the compact sequence set chosen, the simulation effort needed here is still relatively modest and is nowhere near the cutting-edge exascale biomolecular computations.^166–168^

Overall, the present work provides a comprehensive description of DNA and RNA duplexes at the hexanucleotide scale, and proposes and validates a straightforward model to predict shape and harmonic stiffness for an arbitrary sequence with minimum additional assumptions. The model parameters are made freely available, forming a baseline of further research and allowing for a broad range of applications in molecular biology, biophysics, and nucleic acid nanostructure design.

## Supplementary Material





## Data Availability

The model parameters, heatmap visualizations, as well as the in-house scripts to generate a minimal sequence including all *k*-mers, and to analyze the MD data, are available at Zenodo, https://zenodo.org/records/15575888. The raw, water-stripped MD data of set107 has been uploaded to Lexis, https://portal.lexis.tech (project exa4 mind_wp4).

## Notes

Supporting methods: sequence design; cutoff optimization; Supporting results: bending and twisting static disorder; Supporting figures: eigenvalues of the stiffness matrix for various base–base interaction ranges—DNA; eigenvalues of the stiffness matrix for various base–base interaction ranges—RNA; hexameric context dependence for selected RNA tetramers; histograms of major groove widths; histograms of major groove stiffness; histograms of stretch modulus, twist rigidity, and TS coupling; histograms of bending and twisting persistence lengths; semilog plots to infer dynamic bending persistence lengths; semilog plots to infer twist persistence lengths; semilog plots to infer static bending and twisting persistence lengths; eigenvalues of the MD-derived and the assembled stiffness matrices; eigenvectors of the MD-derived and the assembled stiffness matrices; cutoff optimization for DNA; cutoff optimization for RNA; correlations between MD-derived and model-generated elastic constants; correlations between MD-derived and model-generated persistence lengths; intra-basepair coordinates of the Dickerson dodecamer—prediction and experiment; inter-basepair coordinates of the Dickerson dodecamer—prediction and experiment; deformation energy of random sequence and polyA in nucleosome; major groove widths of DNA A-tract and RNA AU-tract; structure of DNA AT-tracts; logarithm of the survival function; distributions of base-pair opening times; distributions of base-pair opening times—long opening events; base-pair opening along a DNA and analogous RNA MD trajectory; structure of broken A-U pairs; probability density of shear in an unfiltered and filtered trajectory; noncanonical DNA structures; effect of the B-like sugar flip in RNA on twist and propeller; Supporting tables: the set52 sequences comprising all pentamers—DNA; the set52 sequences—RNA; the set14 DNA and RNA sequences comprising all tetramers; absolute errors of coordinate means for first and second half of MD trajectory; relative errors of coordinate stiffnesses for first and second half of MD trajectory; relative errors of global stiffness constants for first and second half of MD trajectory; absolute differences between coordinate means of duplicate hexamers; relative differences between coordinate stiffnesses of duplicate hexamers; means and std of equilibrium coordinates for all RNA dimer sequences; errors on predicted intra-bp coordinates and minor groove widths wrt set52 MD data; errors on predicted inter-bp, helical coordinates and major g. widths wrt set52 MD data; relative errors on predicted coordinate stiffnesses wrt set52 MD data; deformation energy of A-tracts and control sequence threaded through the nucleosome; Supporting references (PDF)
A txt file listing the s107 sequence set (TXT)
