# Correction to “Sequence-Dependent Shape and Stiffness of DNA and RNA Double Helices: Hexanucleotide Scale and Beyond”

**DOI:** 10.1021/acs.jcim.5c02218

**Published:** 2025-10-01

**Authors:** Pavlína Slavníková, Marek Cuker, Eva Matoušková, Ivan Čmelo, Marie Zgarbová, Petr Jurečka, Filip Lankaš

In Table 2 of the original paper, the MD standard deviations of the basepair-step coordinates whose averages are exactly zero were missing. The corrected table is provided below. The title and footnote of the table remain unchanged.

**Table 2:** 

Middle dimer	Source	Shift [Å]	Slide [Å]	Rise [Å]	Tilt [°]	Roll [°]	Twist [°]
CG	Olson	0	0.36	3.34	0	6.4	34.3
	MD	0 ± 0.24	0.24 ± 0.12	3.33 ± 0.12	0 ± 1.40	6.16 ± 1.04	34.31 ± 3.11
CA	Olson	–0.06	0.18	3.33	0.0	5.6	35.1
	MD	–0.23 ± 0.20	0.15 ± 0.17	3.29 ± 0.12	–0.11 ± 1.13	6.70 ± 0.98	33.13 ± 3.28
TA	Olson	0	0.20	3.35	0	2.3	37.5
	MD	0 ± 0.28	0.29 ± 0.17	3.25 ± 0.10	0 ± 1.22	5.26 ± 0.87	34.51 ± 2.46
AG	Olson	–0.03	–0.34	3.30	–0.4	3.6	32.2
	MD	–0.32 ± 0.19	–0.37 ± 0.21	3.37 ± 0.09	–2.70 ± 0.62	3.93 ± 1.09	33.46 ± 1.83
GG	Olson	–0.01	–0.33	3.36	0.0	4.9	33.2
	MD	–0.20 ± 0.24	–0.25 ± 0.31	3.45 ± 0.10	–0.06 ± 1.08	4.85 ± 0.76	34.90 ± 1.65
AA	Olson	0.02	–0.27	3.24	0.0	–0.2	35.1
	MD	–0.36 ± 0.19	–0.06 ± 0.16	3.31 ± 0.05	–2.27 ± 0.54	1.63 ± 0.80	35.99 ± 1.44
GA	Olson	–0.02	–0.11	3.28	–0.1	1.9	36.3
	MD	–0.45 ± 0.17	0.23 ± 0.17	3.32 ± 0.06	–1.08 ± 0.95	2.22 ± 0.75	37.74 ± 1.81
AT	Olson	0	–0.69	3.22	0	0.1	30.7
	MD	0 ± 0.10	–0.71 ± 0.06	3.24 ± 0.05	0 ± 0.37	–0.53 ± 0.71	30.70 ± 1.36
AC	Olson	0.00	–0.64	3.26	0.2	1.7	31.9
	MD	0.19 ± 0.17	–0.49 ± 0.13	3.29 ± 0.08	–0.40 ± 0.77	0.22 ± 0.58	32.69 ± 1.88
GC	Olson	0	–0.45	3.29	0	2.7	33.3
	MD	0 ± 0.20	–0.28 ± 0.17	3.35 ± 0.08	0 ± 0.96	0.20 ± 0.39	36.60 ± 1.80
Avg	Olson	–0.01 ± 0.02	–0.21 ± 0.36	3.30 ± 0.05	–0.03 ± 0.15	2.90 ± 2.22	33.96 ± 2.10
	MD	–0.14 ± 0.20	–0.13 ± 0.35	3.32 ± 0.06	–0.66 ± 1.02	3.06 ± 2.66	34.40 ± 2.05

Furthermore, the subsection title “Structural Dynamics” should be upgraded by one level, to be at the same level as, for example, “A Predictive Model of Sequence-Dependent Shape and Stiffness”.

