# Supplementary material for: Sequence-Dependent Shape and Stiffness of DNA and RNA Double Helices: Hexanucleotide Scale and Beyond
Source: J Chem Inf Model. 2025 Aug 25;65(17):9208–29. doi: 10.1021/acs.jcim.5c00576 (PMC12421674; doi:10.1021/acs.jcim.5c00576)
Supplement: Supplementary file 1 [file ci5c00576_si_001.pdf]

SUPPORTING INFORMATION

Sequence-dependent shape and stiffness of DNA and RNA double helices: hexanucleotide scale and beyond

Pavlína Slavníková[1], Marek Cuker[1], Eva Matoušková [1], Ivan Čmelo [1,2,3], Marie Zgarbová [4], Petr Jurečka [4], Filip Lankaš [1*]

[1] Department of Informatics and Chemistry, University of Chemistry and Technology Prague, 166 28 Prague, Czech Republic

[2] CZ-OPENSCREEN: National Infrastructure for Chemical Biology, Faculty of Chemical Technology, University of Chemistry and Technology Prague, 166 28 Prague, Czech Republic

[3] Molecular Design Group, School of Chemical Sciences, Dublin City University, D09 V209 Glasnevin, Ireland

[4] Department of Physical Chemistry, Faculty of Science, Palacký University, 771 46 Olomouc, Czech Republic

*Corresponding author, filip.lankas@vscht.cz

Supporting methods

Sequence design

To obtain the pentamer sequence, a basic depth-first search approach was developed and implemented in-house, in the form of a Python script. While there are already several available tools for *k*-mer optimal sequence generation (1), adding a simple, easily modifiable function to a pre-existing Python workflow was a matter of flexibility and convenience. The script, after being given a seed sequence (e.g. AAAAA), attempts to extend the sequence by another base. If the appended base forms a new *k*-mer, the addition is accepted and the script advances one base further. If the appended base does not form a new *k*-mer, the script attempts to change it for another base and repeats the test. If the script exhausted all bases to no avail, the script backs up one base, changes it and repeats the test, thus in effect backing out of the unproductive branch. To expedite the process, the script also maintains a memory of encountered unproductive branches in the form of a hash set. The hashes encode the *k*-mer interface (i.e. the last (*k*-1)-mer) and all missing *k*-mers. Whenever the script encounters a *k*-mer interface and missing *k*-mer set that corresponds to a known unproductive branch, that branch is immediately skipped. Optionally, the script also features a configurable iteration limit. Upon exhausting a predefined number of attempts to extend the sequence in an optimal manner (i.e. every added base introduces a novel *k*-mer), the script reverts to the best encountered partial solution, extends it by two or more bases to directly incorporate the best-fitting missing *k*-mer, and continues its search from that point. This allows a configurable trade-off between search thoroughness and speed.

Beyond the required pentamer sequence, the script was also tested for other *k*-mer sizes, from dimers to nonamers. For odd-numbered *k*-mers, the developed depth-first search script seems to perform well, producing optimal sequences. However, this approach performed significantly worse for even-numbered *k*-mers, likely due to their symmetry (possible self-complementarity) resulting in a less favorable search tree topology. The sub-optimal solutions for even-numbered *k*-mers, namely tetramers, hexamers and octamers, are respectively 4,1%, 1.9% and 1% less efficient than sequences produced by the dedicated ShortCAKE tool by Orenstein and Shamir [https://acgt.cs.tau.ac.il/sho] (1). Thus, to obtain the hexamer sequence, the ShortCAKE tool was used, as it provides optimal-length (i.e. shortest possible) sequences both for odd and even-numbered *k*-mers.

Cutoff optimization

To optimize the cutoff, we cannot use the hexameric set107, since the matrix blocks were deduced from there and, if they are assembled back, they simply give the same banded matrices they were cut out from (save possibly of the duplicate hexamers, Methods). We cannot use the pentameric set52 either, since this is the validation set. Thus, we made use of the set of DNA and RNA oligomers containing all tetramers (set14), of which the DNA version has been published (2) and the RNA version was simulated for this work. The target quantities were the global material constants (stretching and twisting stiffness, TS coupling, dynamic bending and twisting persistence lengths). For a given cutoff, we assembled the stiffness matrices for the set14 sequences, applied the cutoff (main text), and prepared the corresponding structural ensembles by generating the multi-dimensional normally distributed intra-base pair and step coordinates. The inner duplex part of set14 has 14 bp, so the dimensionality is 12*14 - 6 = 162. Generating multidimensional random variables falls into the broad category of Monte Carlo methods, although no Metropolis algorithm was used here. We then computed the material constants for this model data as well as for the actual MD data of set14. We repeated the procedure for various cutoffs and chose the one for which the Pearson correlation coefficients between the material constants computed in both ways were maximal. It has turned out that the correlations are weak functions of the cutoff (Fig. S13, S14), enabling us to choose the optimal values with little numerical sensitivity.

Supporting results

Bending and twisting static disorder

Static structural disorder in DNA and RNA double helices is generated due to the sequence-dependent variability of the equilibrium, or static, structure. A single, short oligomer simply has a particular static structure and one cannot speak about a disorder. However, the structure of a very long stretch of the double helix with random sequence, or a large ensemble of shorter helices with variable sequences, may exhibit statistical properties. It has long been known that the static disorder with respect to bending may be quantified by the static bending persistence length l_s, defined by the relation $\langle \boldsymbol{t}_i \cdot \boldsymbol{t}_0 \rangle = \exp(-l/l_s)$, where the brackets now denote the average over the static structural ensemble (3). A disadvantage of this definition is that the sequence-averaged structure over which the static disorder is superimposed is assumed straight, which may not be the case. This holds especially for RNA helices where the A-form implies a significant inclination of the base pairs with respect to the helical axis, resulting in periodic changes of the angle between the base-pair normals with the helical repeat. To deal with this problem, we propose to compute

the static bending p.l. from the definition entirely analogous to the dynamic one (Eq. 4 of the main text), but now considering the static structural ensemble in place of the MD trajectory snapshots. In this way, the sequence-averaged static structure is factored out.

Just as the static disorder with respect to bending, one may study the disorder with respect to twisting which, we believe, has not been examined so far. We again use the definition entirely analogous to the twist (dynamic) persistence length (Eq. 5 of the main text) to define the twist static persistence length, considering the static ensemble instead of an ensemble of MD snapshots.

Here we infer the static bending and twisting persistence lengths based on the hexanucleotide structural data from our s107 MD set. To do so, we generate 10^5 random DNA sequences and the same number of random RNA sequences, each 500 bp long, and assign a static structure to each of them based on our hexameric equilibrium coordinate data to obtain the structural ensemble, then use Eq. 4 and 5 (main text) to infer the persistence lengths. The periodic oscillations are nicely factored out, and the semilog plots are very close to linear (Fig. S10). We find that the static disorder based on the MD data, both with respect to bending and twisting and both for DNA and RNA, is very weak. The computed DNA static bending persistence length is 963 nm, the RNA one equals to 890 nm. The twist disorder we found is even weaker, the DNA and RNA half static twist persistence lengths being 1647 and 3687 nm, respectively. Thus, the dynamic persistence lengths in Table 3 (main text) should be also understood as the total ones (we are not aware of any relation for the twist p.l. analogous to $1/l_{tot} = 1/l_d + 1/l_s$, but it is reasonable to assume that the static and thermal noise superimpose in some way).

List of supporting figures

Fig. S1	Eigenvalues of the stiffness matrix for various base-base interaction ranges – DNA.
Fig. S2	Eigenvalues of the stiffness matrix for various base-base interaction ranges – RNA.
Fig. S3	Hexameric context dependence for selected RNA tetramers.
Fig. S4	Histograms of major groove widths.
Fig. S5	Histograms of major groove stiffness.
Fig. S6	Histograms of stretch modulus, twist rigidity, and TS coupling.
Fig. S7	Histograms of bending and twisting persistence lengths.
Fig. S8	Semilog plots to infer dynamic bending persistence lengths.
Fig. S9	Semilog plots to infer twist persistence lengths.
Fig. S10	Semilog plots to infer static bending and twisting persistence lengths.
Fig. S11	Eigenvalues of the MD-derived and the assembled stiffness matrices.
Fig. S12	Eigenvectors of the MD-derived and the assembled stiffness matrices.
Fig. S13	Cutoff optimization for DNA.
Fig. S14	Cutoff optimization for RNA.
Fig. S15	Correlations between MD-derived and model-generated elastic constants.
Fig. S16	Correlations between MD-derived and model-generated persistence lengths.
Fig. S17	Intra-base pair coordinates of the Dickerson dodecamer – prediction and experiment.
Fig. S18	Inter-base pair coordinates of the Dickerson dodecamer – prediction and experiment.
Fig. S19	Deformation energy of random sequence and polyA in nucleosome.
Fig. S20	Major groove widths of DNA A-tract and RNA AU-tract.
Fig. S21	Structure of DNA AT-tracts.
Fig. S22	Logarithm of the survival function.
Fig. S23	Distributions of base-pair opening times.
Fig. S24	Distributions of base-pair opening times – long opening events.
Fig. S25	Base-pair opening along a DNA and analogous RNA MD trajectory.
Fig. S26	Structure of broken A-U pairs.
Fig. S27	Probability density of shear in an unfiltered and filtered trajectory.
Fig. S28	Non-canonical DNA structures.
Fig. S29	Effect of the B-like sugar flip in RNA on twist and propeller.

List of supporting tables

Table S1	The set52 sequences comprising all pentamers – DNA.
Table S2	The set52 sequences – RNA.
Table S3	The set14 DNA and RNA sequences comprising all tetramers.
Table S4	Absolute errors of coordinate means for first and second half of MD trajectory.
Table S5	Relative errors of coordinate stiffnesses for first and second half of MD trajectory.
Table S6	Relative errors of global stiffness constants for first and second half of MD trajectory.
Table S7	Absolute differences between coordinate means of duplicate hexamers.
Table S8	Relative difference between coordinate stiffnesses of duplicate hexamers.
Table S9	Means and std of equilibrium coordinates for all RNA dimer sequences.
Table S10	Errors on predicted intra-bp coordinates and minor groove widths wrt set52 MD data.
Table S11	Errors on predicted inter-bp, helical coordinates and major g. widths wrt set52 MD data.
Table S12	Relative errors on predicted coordinate stiffnesses wrt set52 MD data.
Table S13	Deformation energy of A-tracts and control sequence threaded through the nucleosome.

Supporting figures

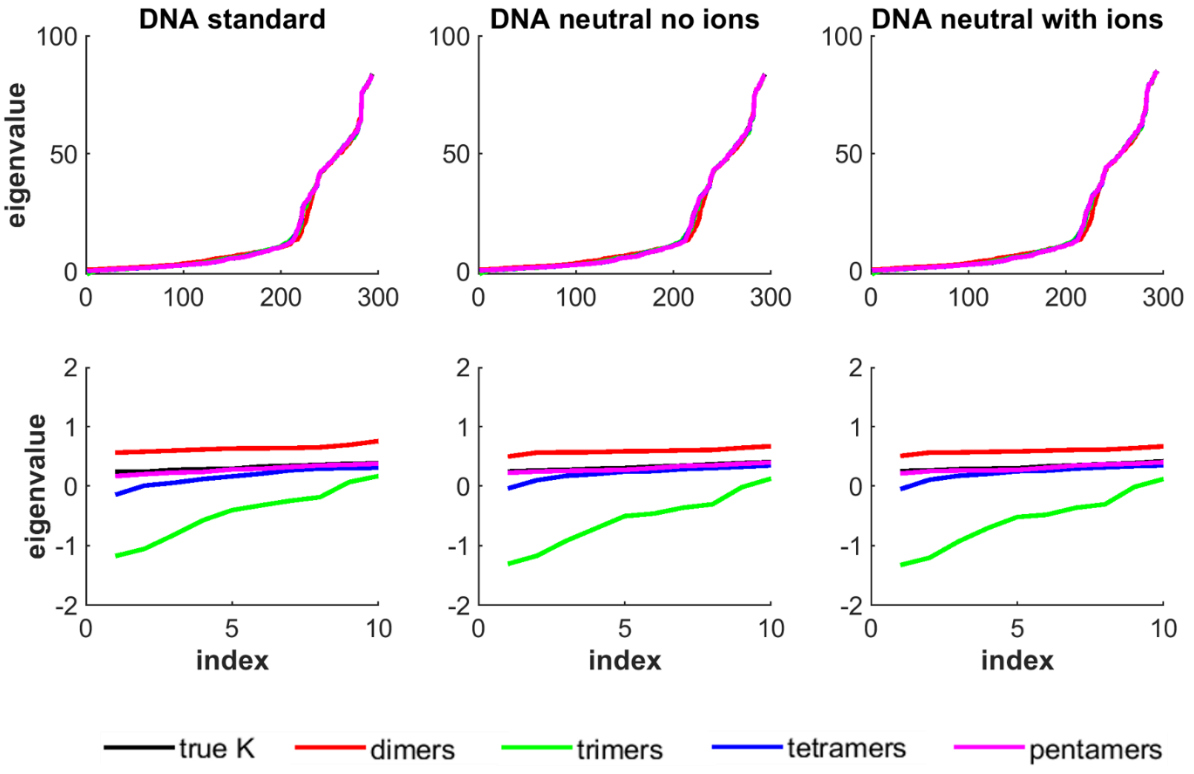

Figure S1. Eigenvalues of the (non-dimensionalized) stiffness matrix for the s0 sequence in its DNA form, with various base-base interaction ranges imposed.

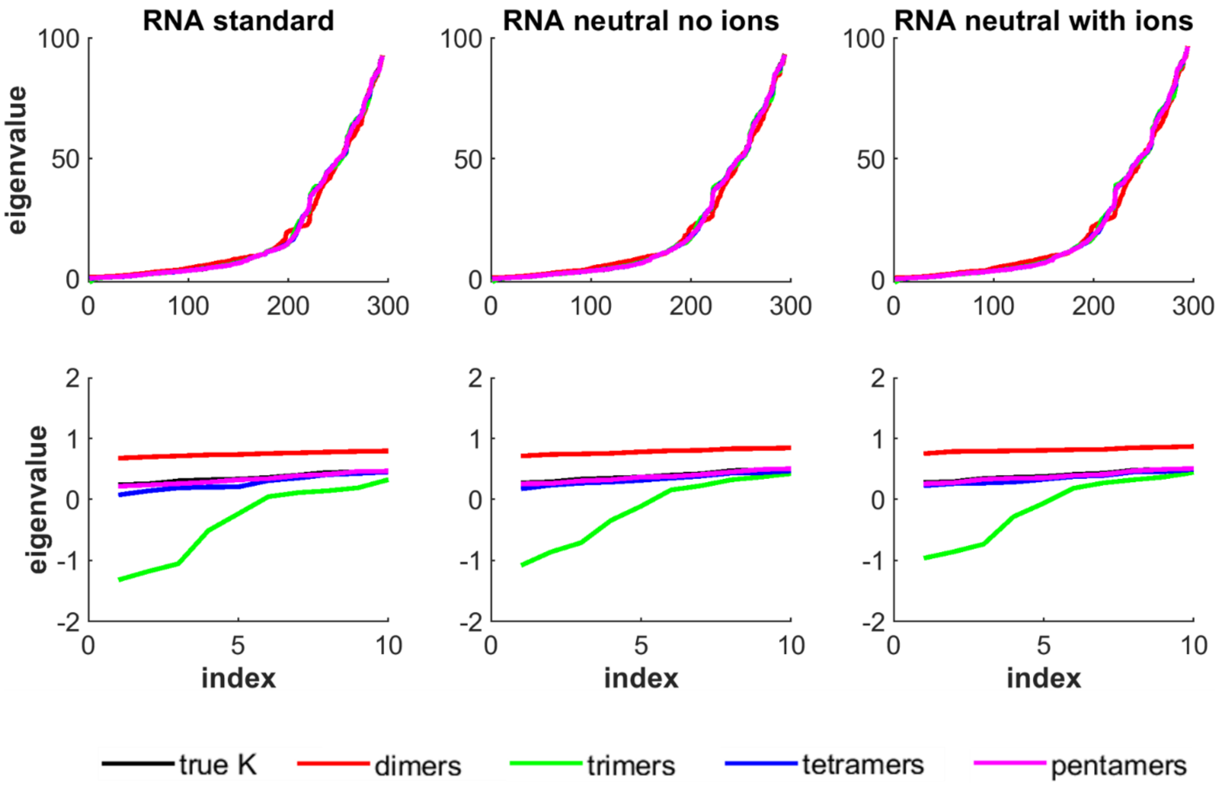

Figure S2. Eigenvalues of the (non-dimensionalized) stiffness matrix for the s0 sequence in its RNA form, with various base-base interaction ranges imposed.

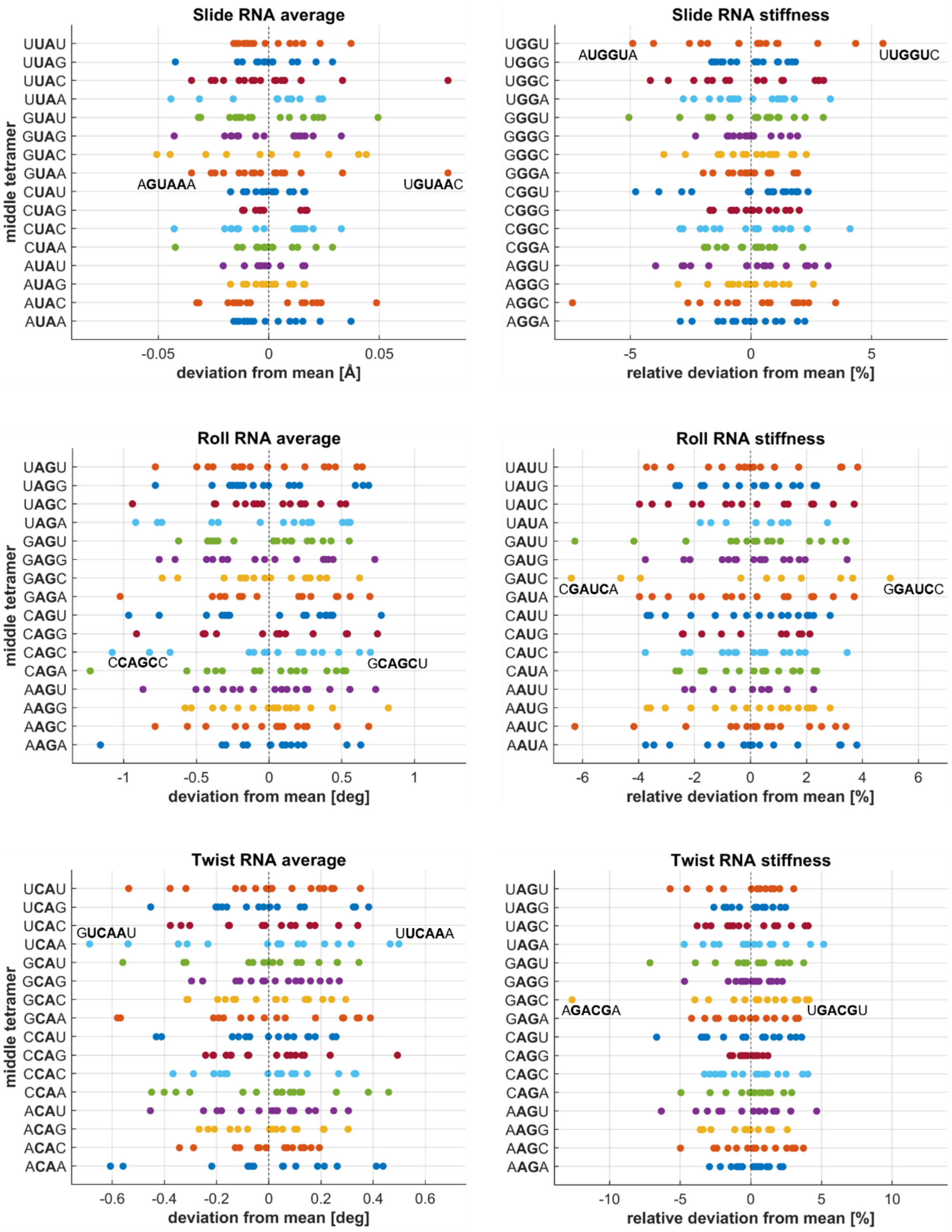

Figure S3. Hexameric context dependence for selected RNA tetramers. Each panel shows all tetramers sharing the same central step. The reference values are averages over all hexameric contexts for the given tetramer. Hexamer-dependent deviations from these values are plotted.

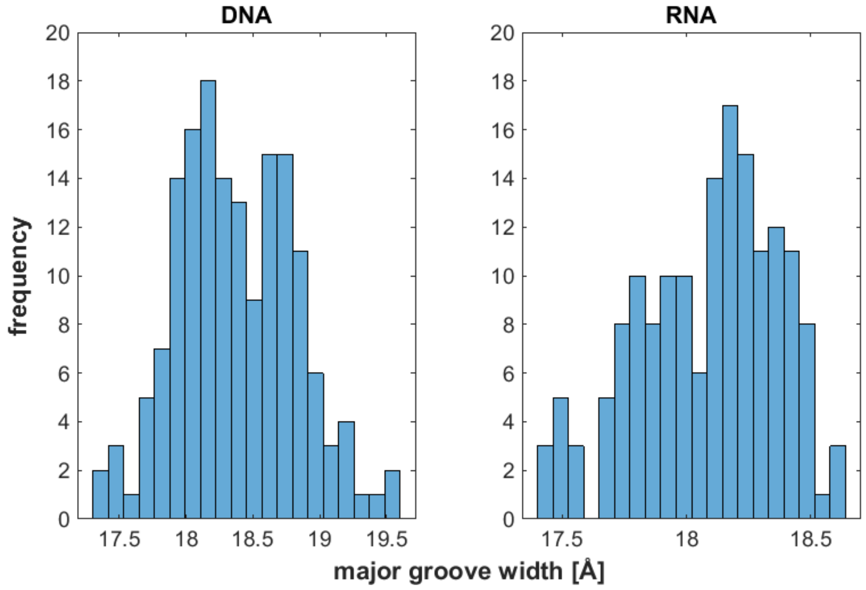

Figure S4. Histograms of major groove width for all the hexanucleotide sequences.

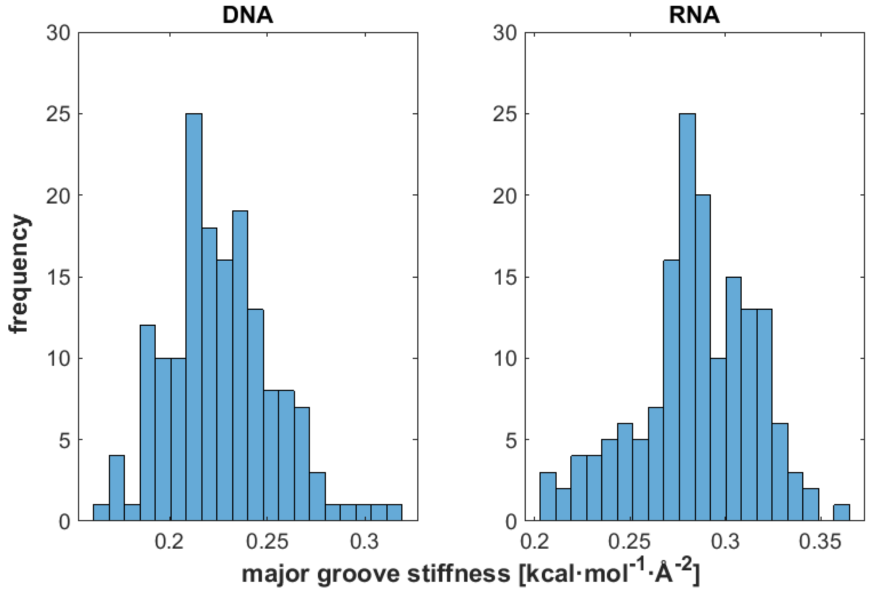

Figure S5. Histograms of major groove stiffness for all the hexanucleotide sequences.

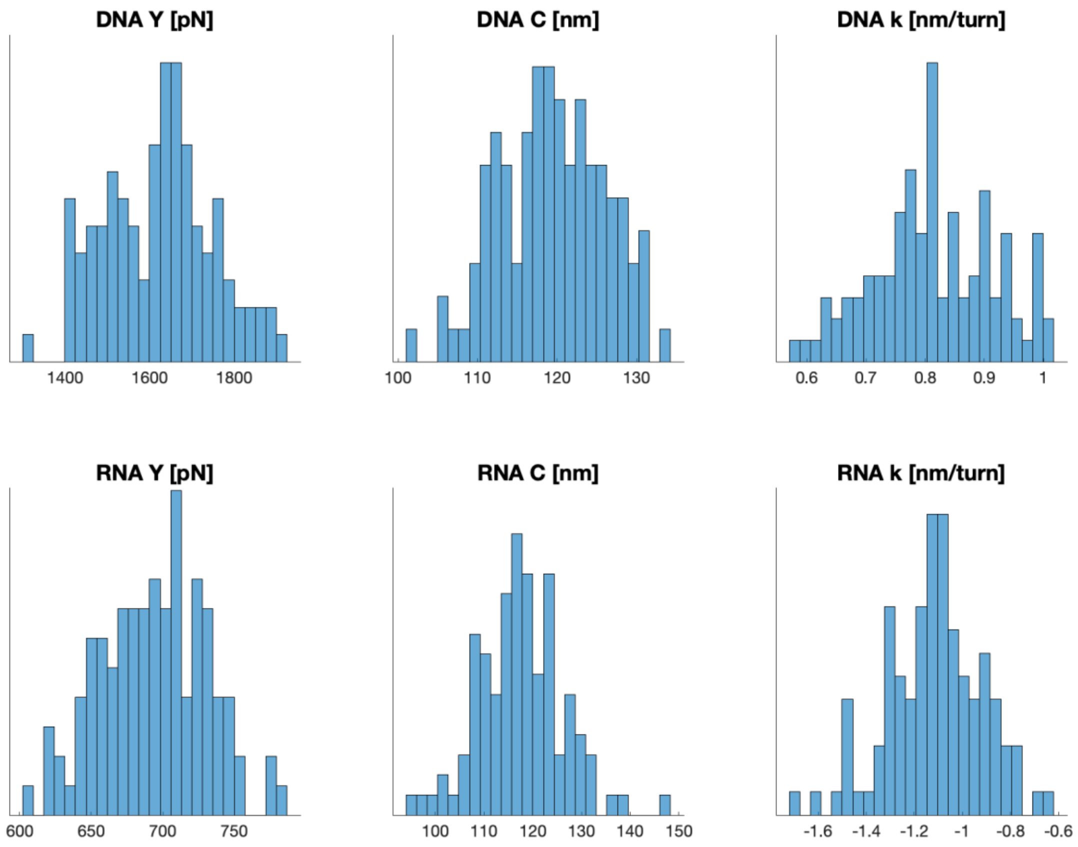

Figure S6. Histograms of the stretch modulus, twist rigidity, and twist-stretch coupling computed for the 107 DNA and 107 RNA oligomers containing all hexanucleotide sequences.

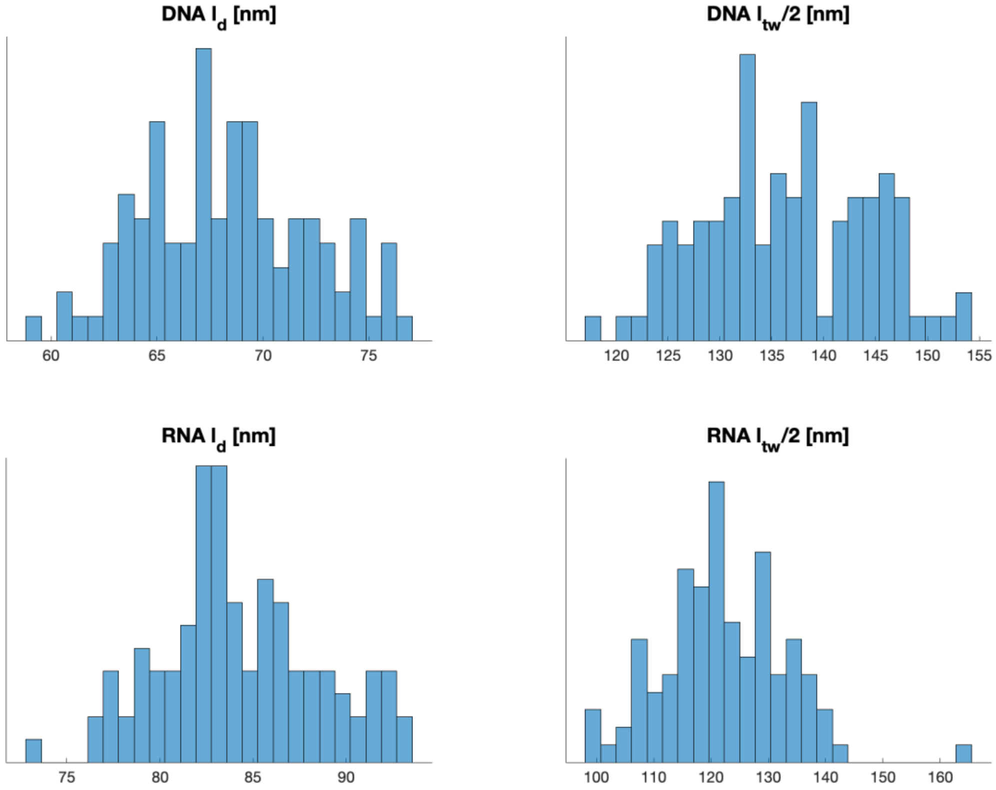

Figure S7. Histograms of bending and half twisting persistence lengths computed for the 107 DNA and 107 RNA oligomers containing all hexanucleotide sequences.

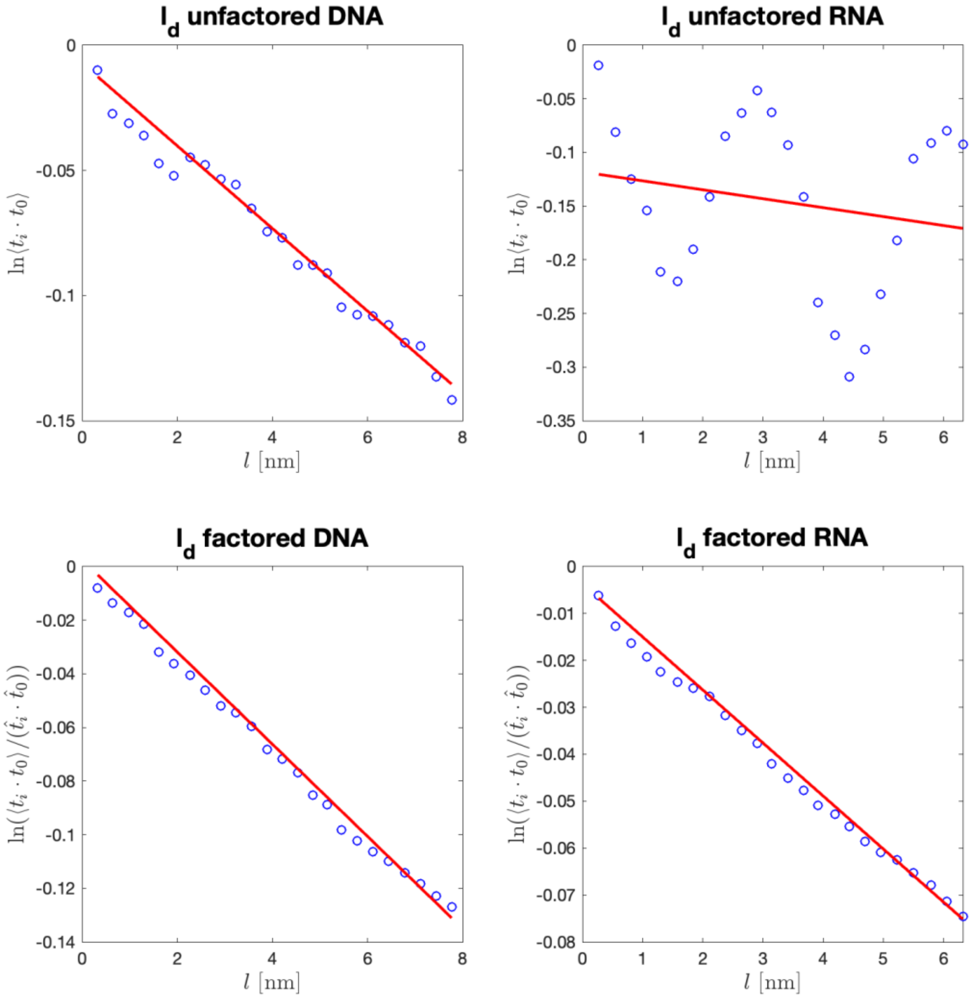

Figure S8. Semilog plots used to infer the dynamic bending persistence length for DNA and RNA duplexes. Data for sequence 37 are shown as an example. Notice the small but discernible shift of the fitting line from the origin, enabled by the introduction of the prefactor α_d in the relation defining the bending persistence length (Eq. 4 of the main text).

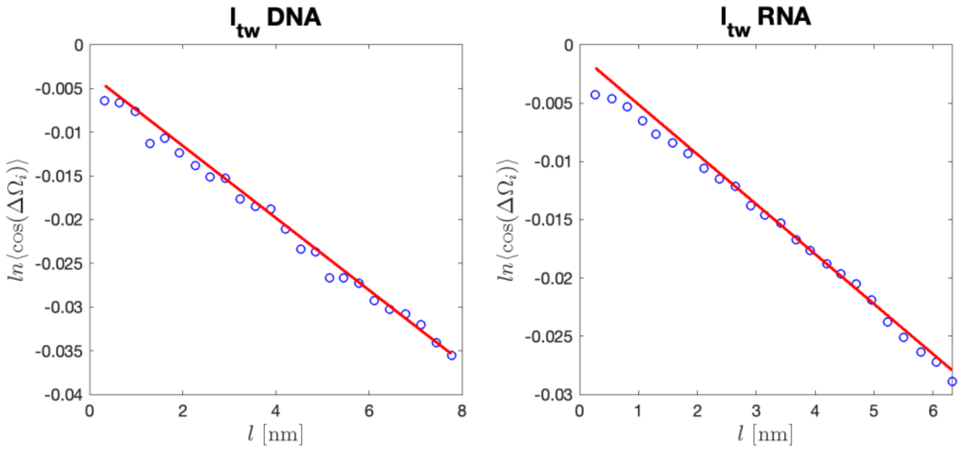

Figure S9. Semilog plots used to infer the twist persistence length. Data for sequence 37 are shown as an example. A clearly visible shift of the fitting line from the origin is captured by the prefactor α_{tw} (Eq. 5 of the main text).

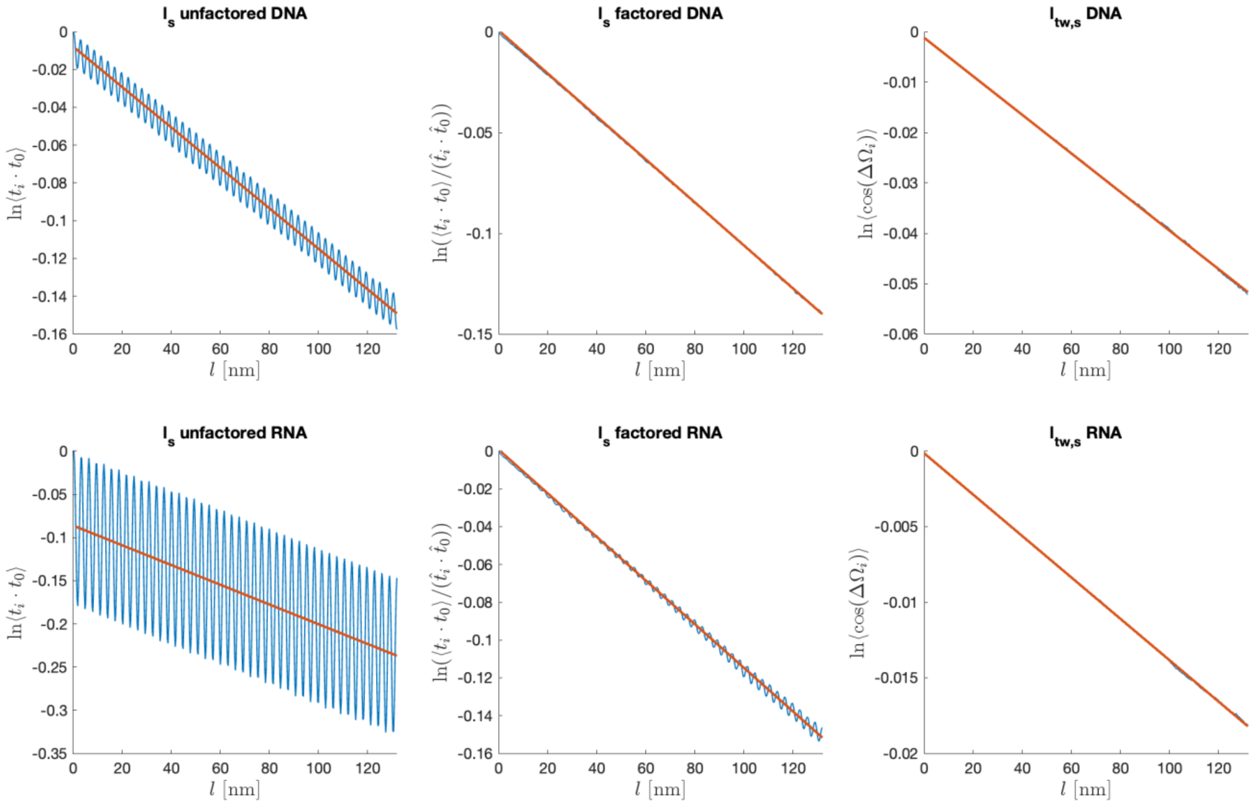

Figure S10. Semilog plots used to infer static bending and twisting persistence lengths.

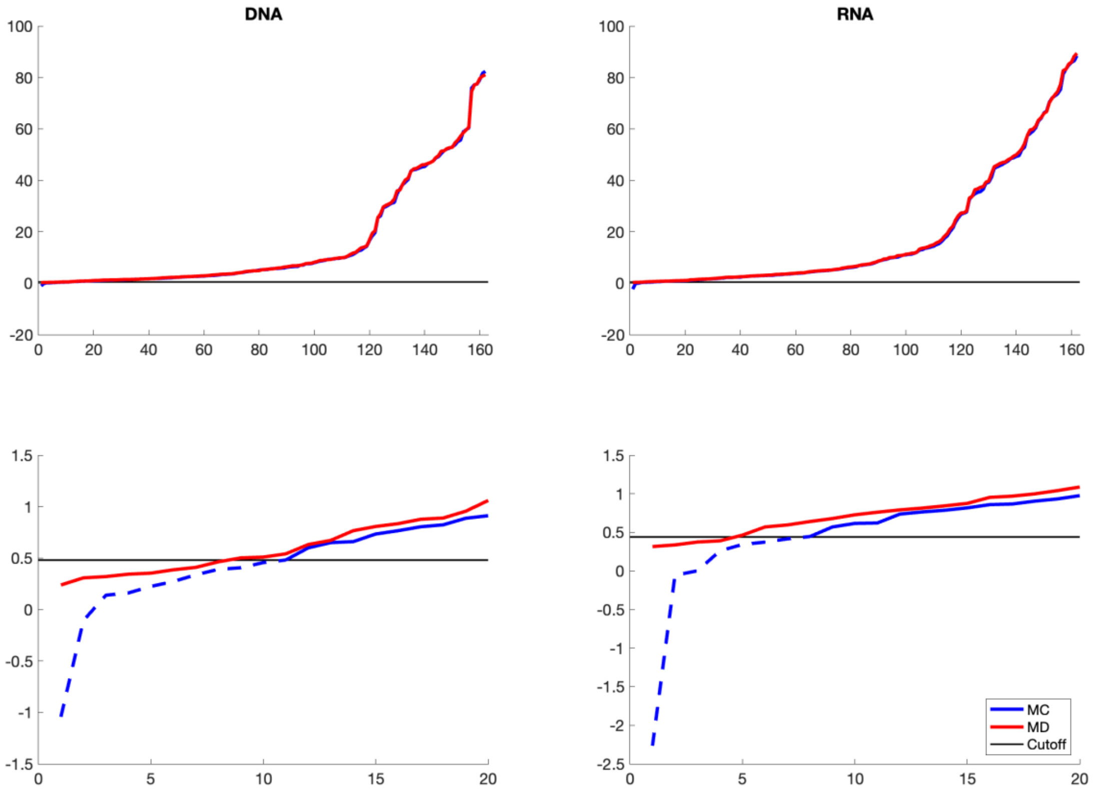

Figure S11. Eigenvalues of the MD-based stiffness matrix (red) and of its approximation by assembled hexameric blocks (blue). The smallest eigenvalues of the approximate matrix are sometimes close to zero or even negative and are replaced by the cutoff value (grey). Sequence 37 of set52 is shown as an example.

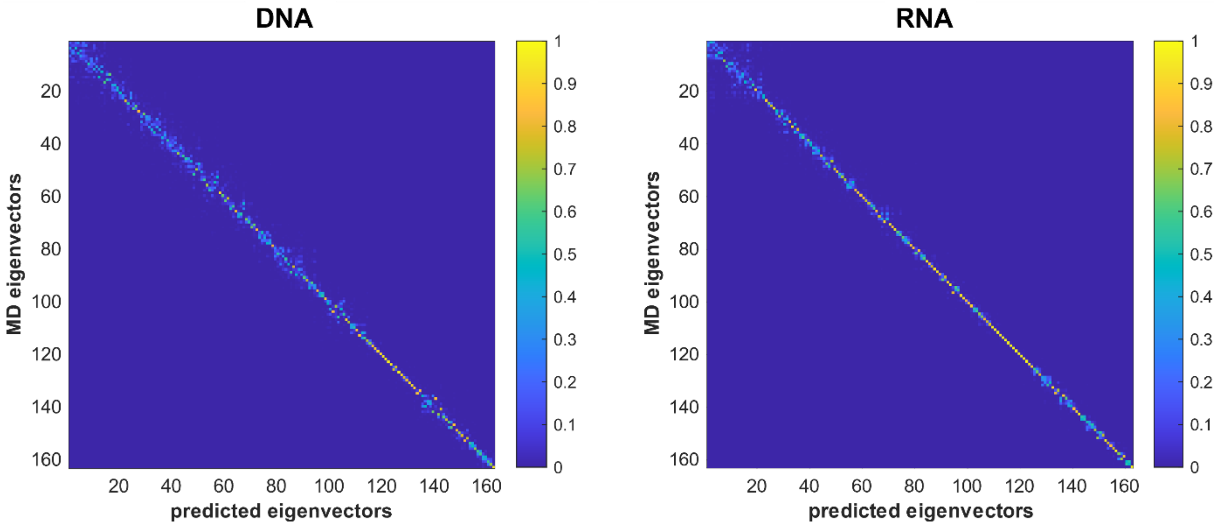

Figure S12. Square dot products between eigenvectors of a stiffness matrix assembled from hexameric blocks and those of the stiffness matrix inferred directly from MD data of the same sequence in the validation set52. Data for sequence 37 of set52.

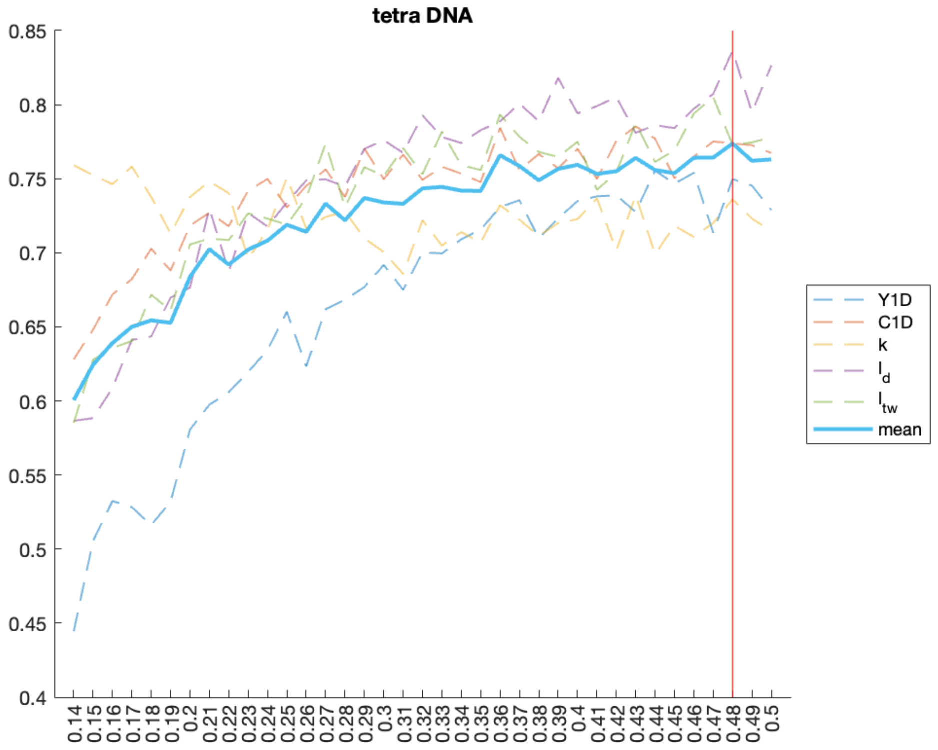

Figure S13. Pearson correlation coefficients between DNA material constants deduced from the set14 MD data and those from model-based structural ensembles (SI Methods) for the same sequences, using the stiffness matrices assembled from hexameric blocks and applying the given cutoff (indicated on the *x* axis). The optimal DNA cutoff value was chosen to be 0.48 (red vertical line).

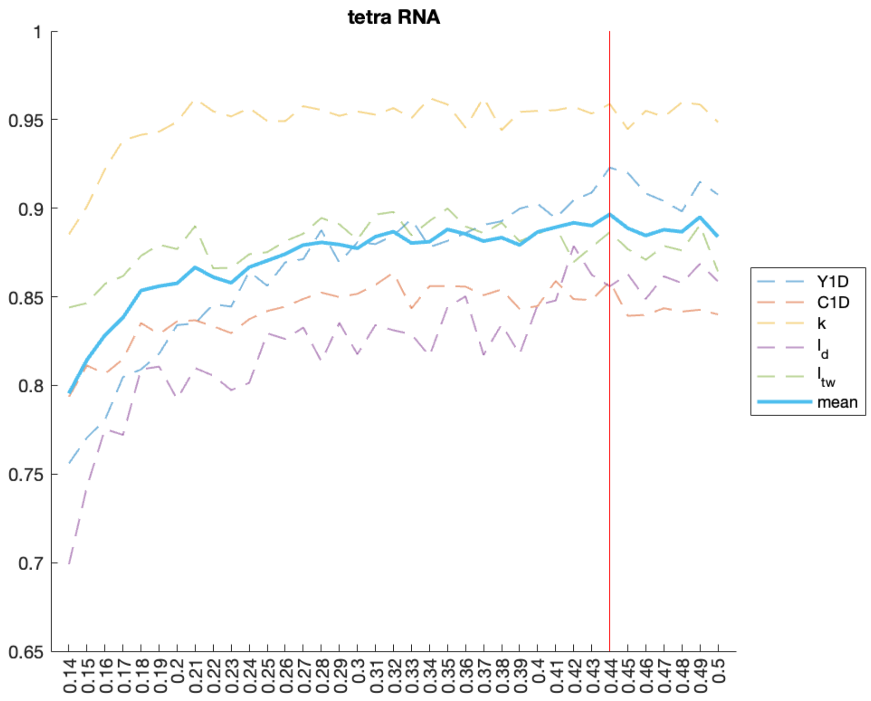

Figure S14. Same as Fig. S13, but for RNA sequences. The optimal RNA cutoff value was chosen to be 0.44 (red vertical line).

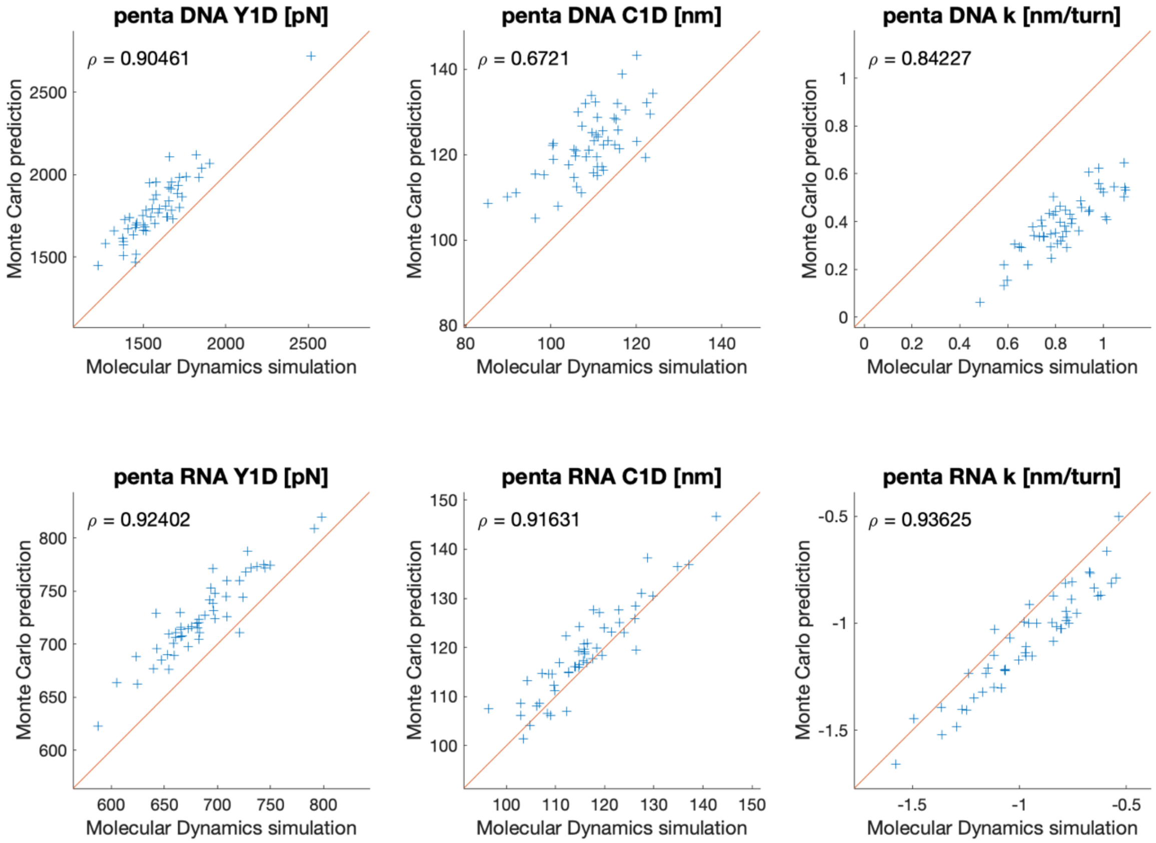

Figure S15. Correlations between elastic constants for the set52 sequences from MD and from structural ensembles generated using the model data. In the latter case, the multidimensional Gaussian distribution of intra-base pair and step coordinates, using the shape and stiffness parameters predicted by the model (with the optimal cutoff applied), was produced for each sequence. The material constants were then deduced from this structural ensemble. The multidimensional random variable generation falls into the broad class of Monte Carlo methods. Pre-defined functions in Matlab were used for this purpose.

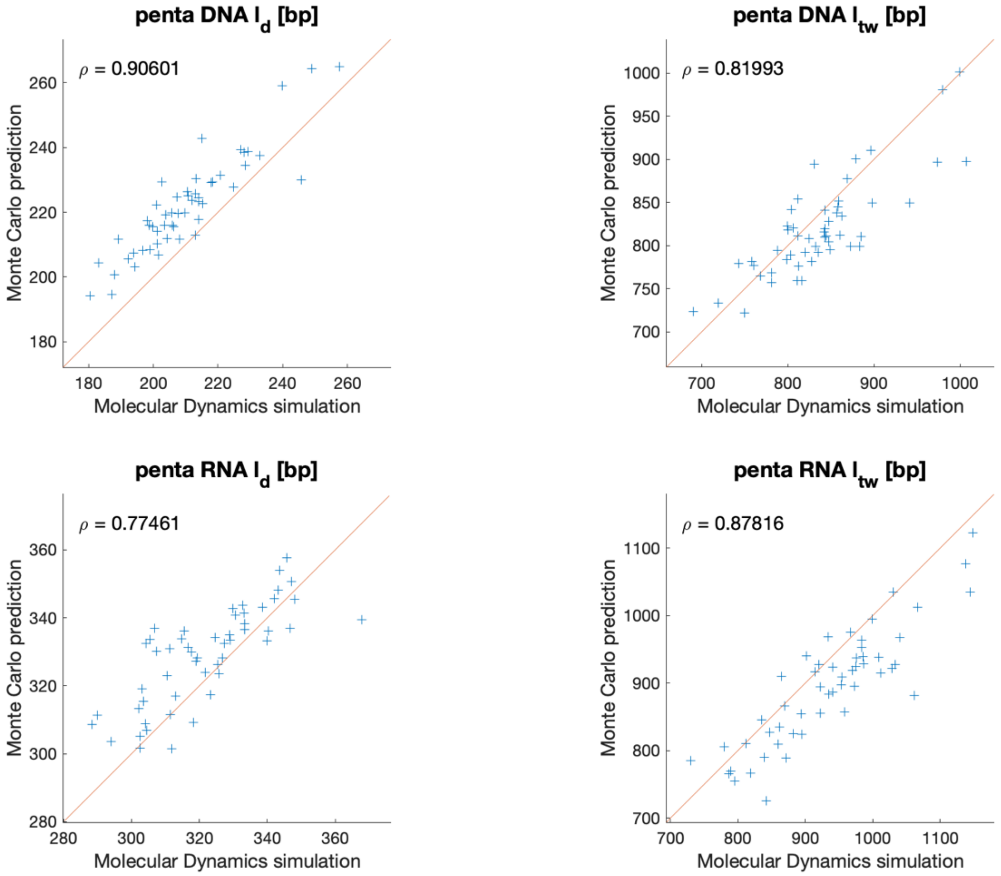

Figure S16. Correlations between persistence lengths for the set52 sequences from MD and from structural ensembles generated using the model predictions (see Fig. S15).

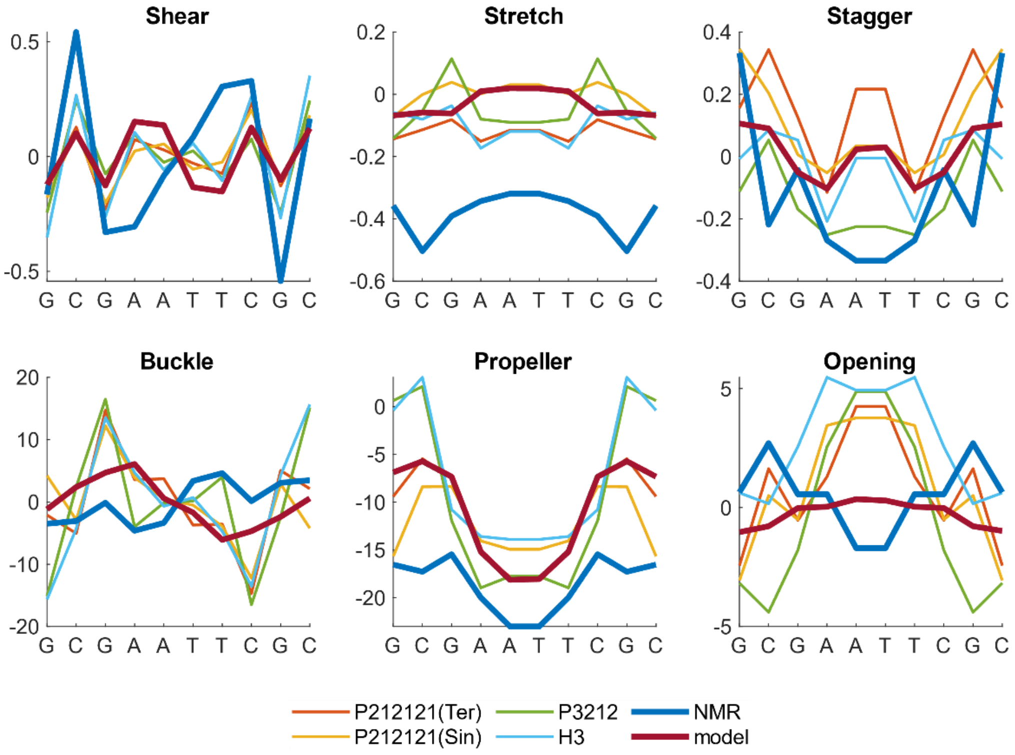

Figure S17. Intra-base pair coordinates for the Dickerson dodecamer. Values predicted by the model (red) are compared to experimental data, labelled as in ref. (4).

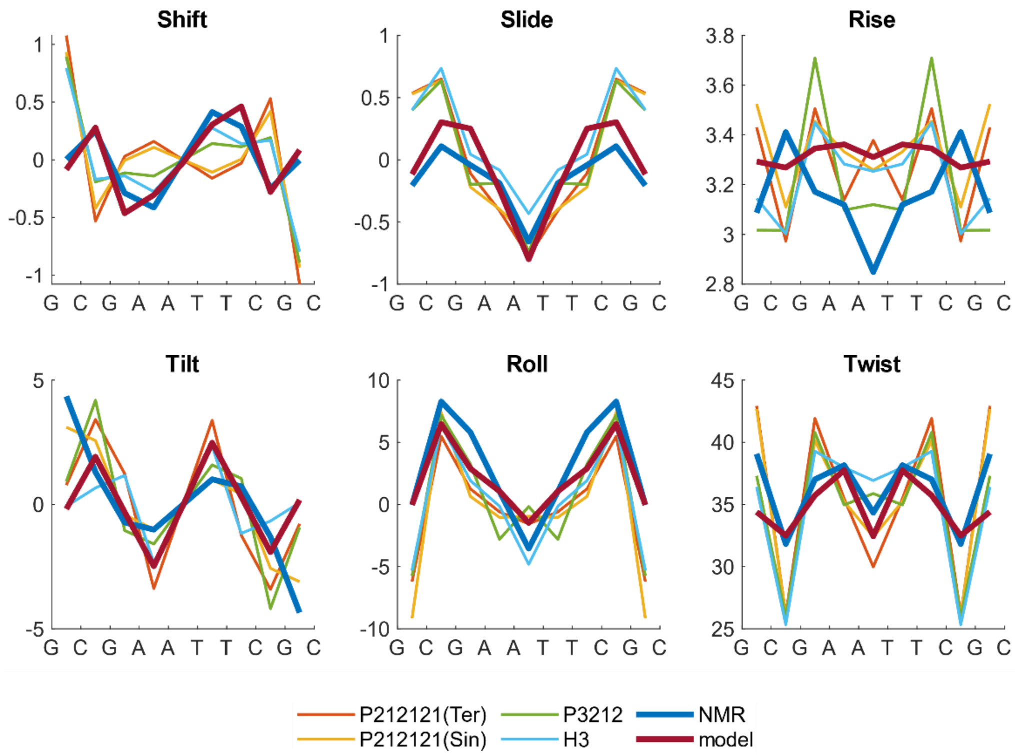

Figure S18. Inter-base pair coordinates for the Dickerson dodecamer. Values predicted by the model (red) are compared to experimental data, labelled as in ref. (4).

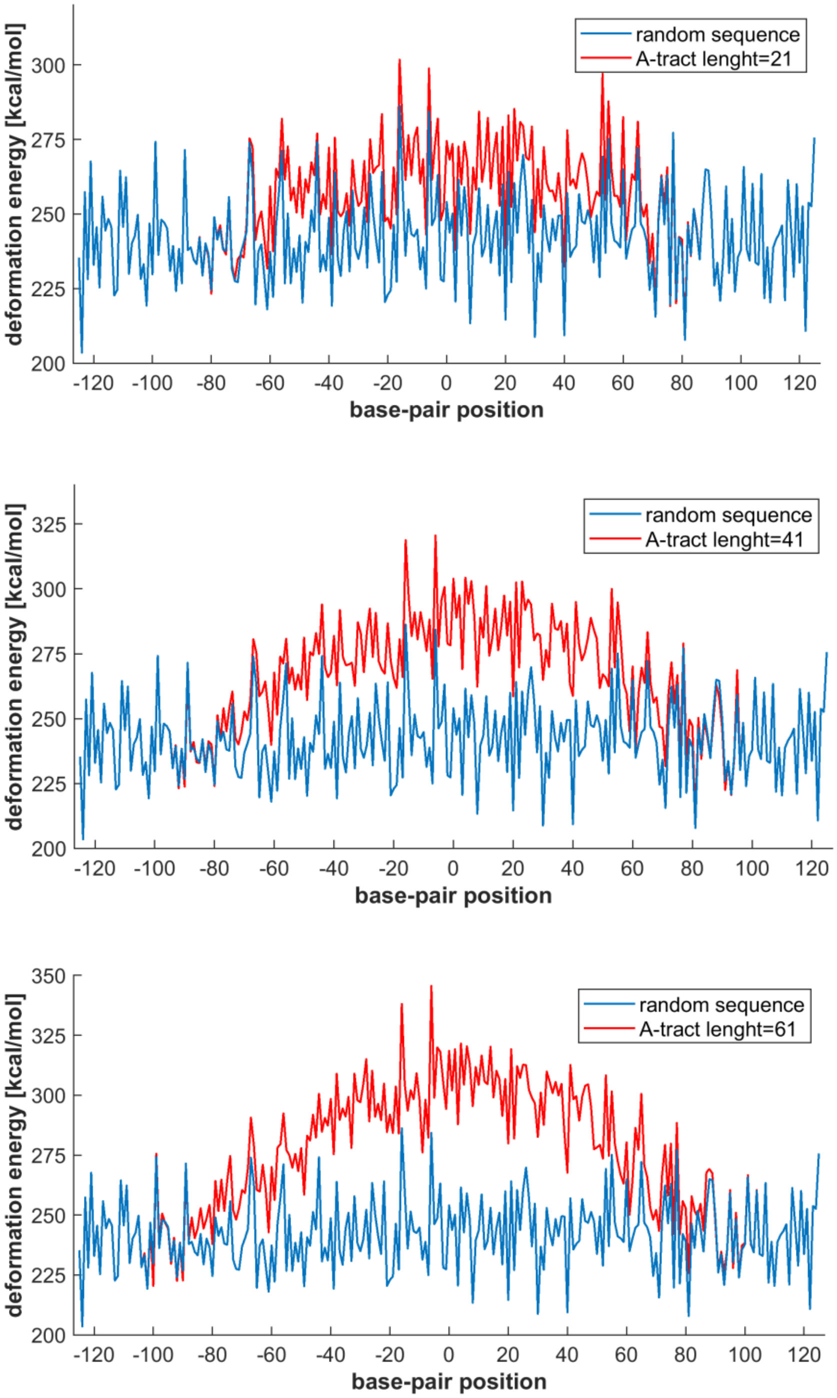

Figure S19. Deformation energy for threading a random sequence (blue), and the same sequence where the central part was mutated to polyA (red) through the 1kx5 nucleosome structure. The roll, twist and slide coordinates, highly conserved among nucleosome structures, were deformed, the remaining coordinates were relaxed to adopt energetically optimal values.

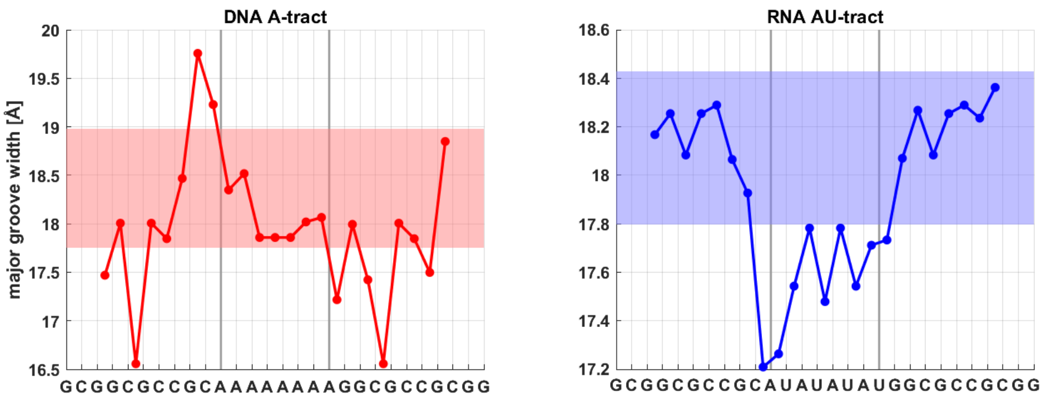

Figure S20. Major groove width profiles of a DNA A-tract and an RNA AU-tract as predicted by our model. The stripes indicate values within one standard deviation from the mean over all hexamers.

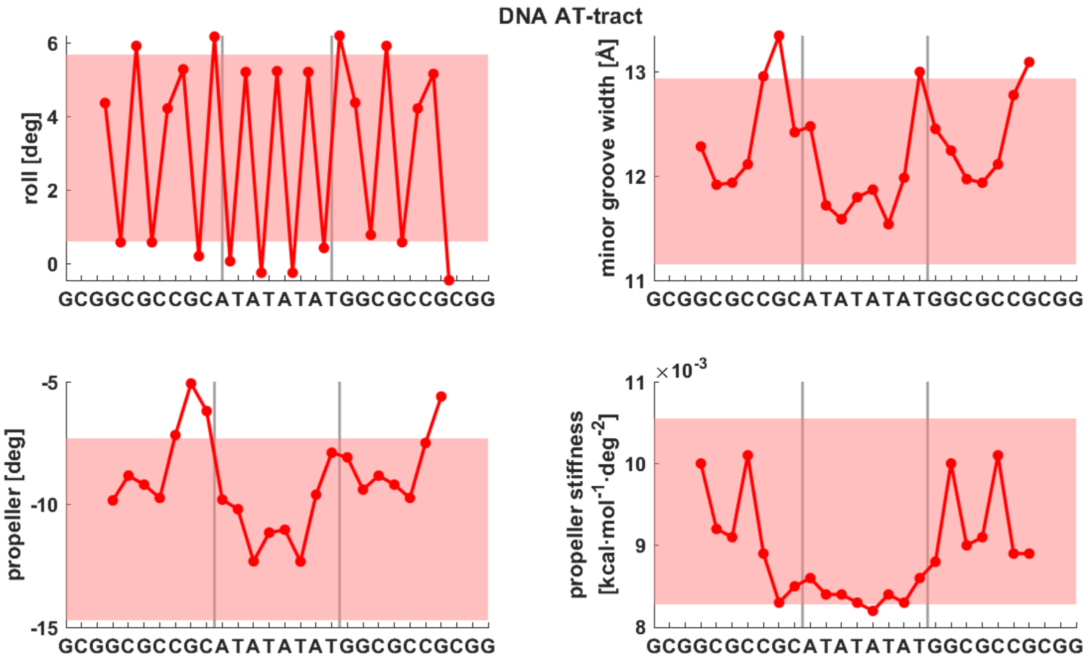

Figure S21. Coordinate profiles for a DNA AT-tract predicted by the model.

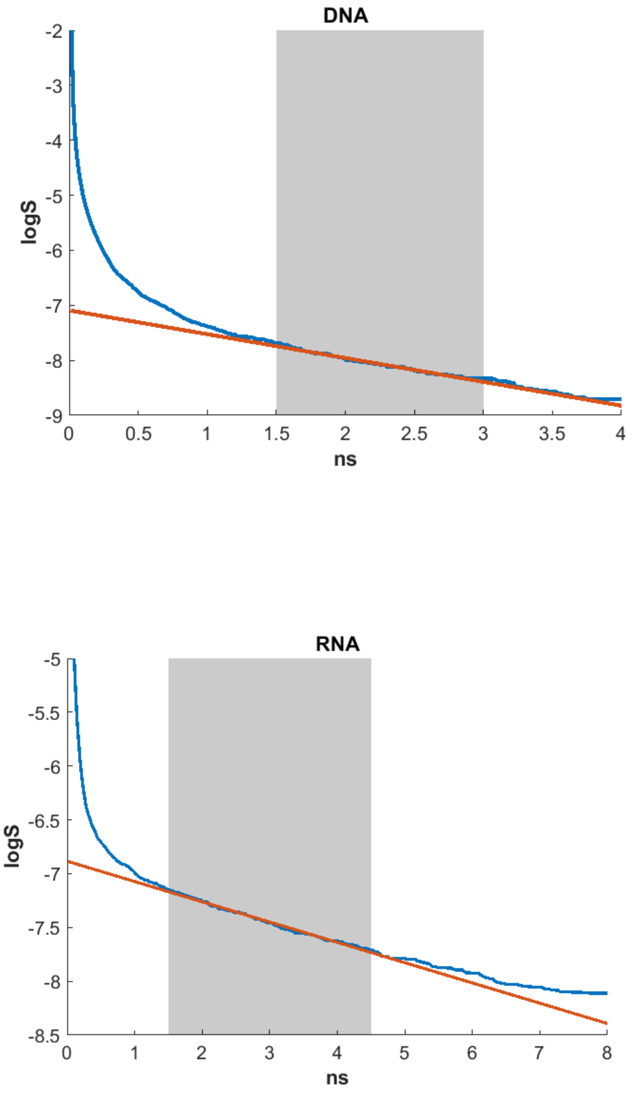

Figure S22. Logarithm of the base-pair opening survival function $S(t)$ vs. time. The visibly linear part within the grey band was fitted with a straight line (red) to obtain the mean opening times.

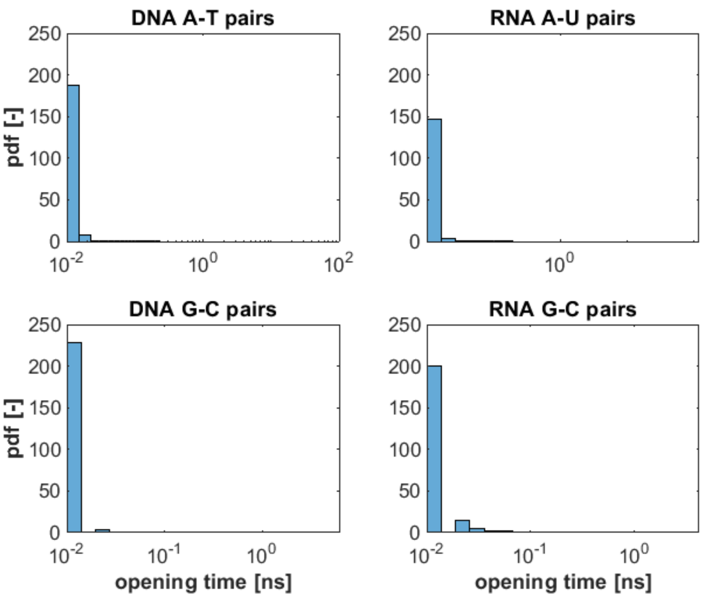

Figure S23. Probability density function of base-pair opening times.

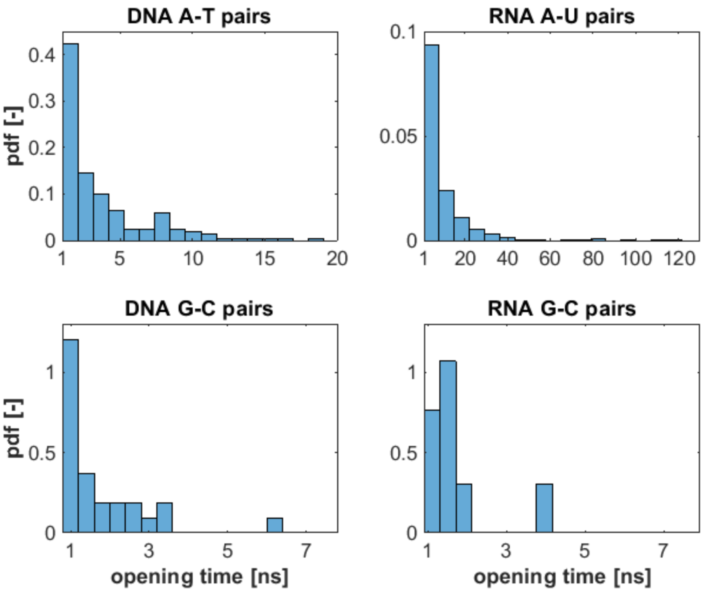

Figure S24. A zoom-in of Fig. S23.

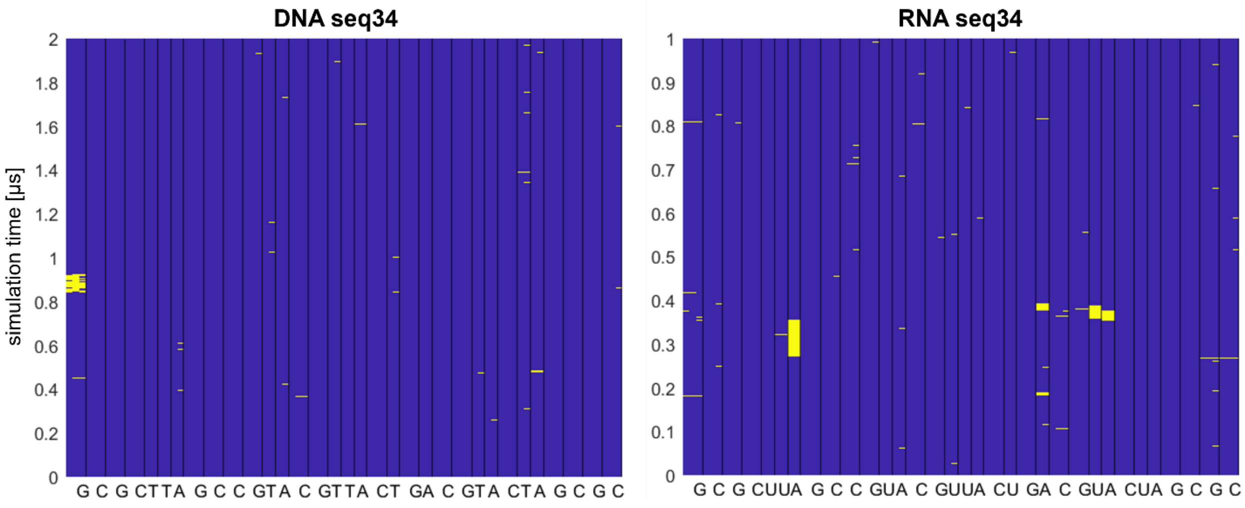

Figure S25. Base pairing dynamics in DNA and RNA duplexes. Long breaks of the RNA A-U pairs (right) have no analogy in DNA (left). A sequence with a typical behavior is shown.

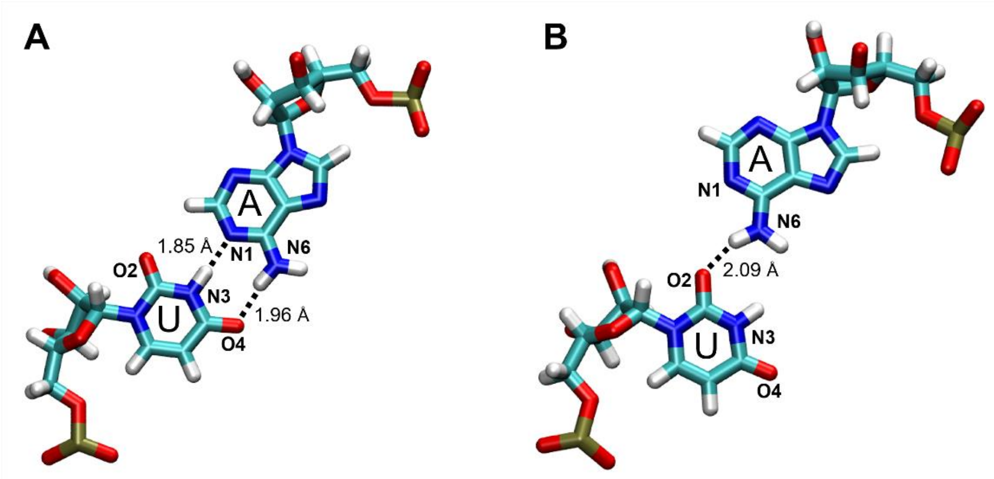

Figure S26. The structure of long-living broken A-U pairs. A typical snapshot is shown.

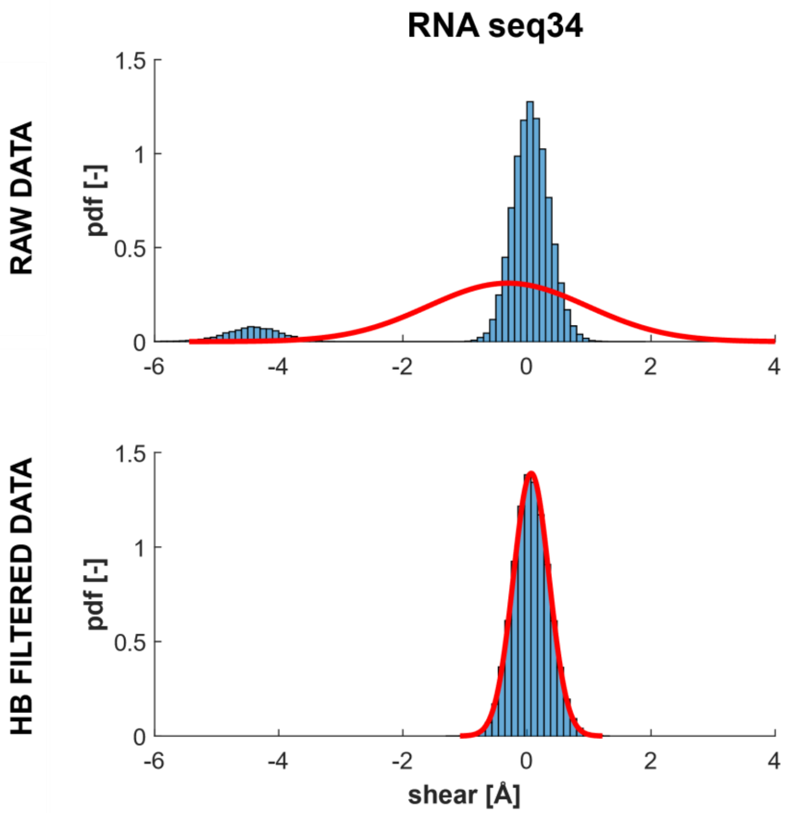

Figure S27. Probability density of the shear coordinate in a pair involved in the long A-U base-pair break (the bp suffering the long break on the left of the RNA panel in Fig. S25 is shown as an example). The Gaussian density with the same mean and standard deviation is also shown (red curves).

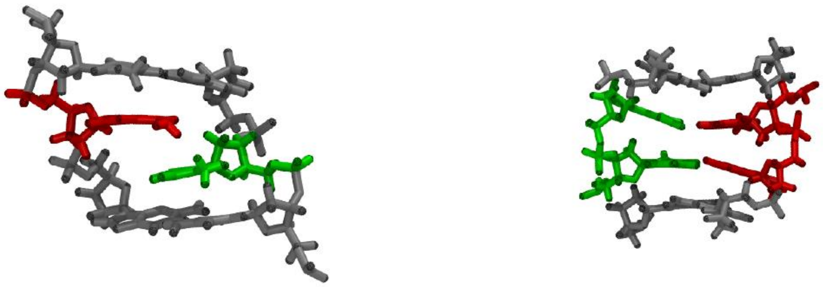

Figure S28. Two types of long-living (> 10 ns) non-canonical structures observed in the 107 DNA simulation set: an inter-strand stack (left, one case) and a ladder-like structure associated with concerted flips of the backbone torsions (right, three cases). Lifetimes 15 – 30 ns.

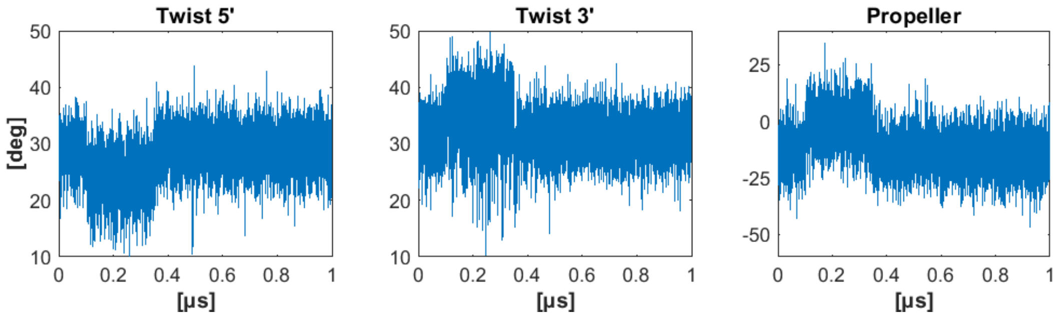

Figure S29. Time series of twist in the RNA steps surrounding the pair with the pucker flipped into the B domain, and the propeller of the pair itself. Significant changes are observed within the time interval of the flip (100 – 350 ns, Fig. 9 of the main text). The sum of the twists still attains the usual value, so that the RNA duplex as a whole is not over- or underwound during the flip.

Supporting tables

Table S1. Simulated oligomers comprising all 512 unique DNA pentamers (set52, DNA).

DNA	5'-------------------3'	DNA	5'-------------------3'
seq1	GCGCAAAATAGCCACTCTGCGC	seq27	GCGCGGAAACGCTTTTTCGCGC
seq2	GCGCCTCTCTTCATAGTTGCGC	seq28	GCGCTTTCTTGTGCATACGCGC
seq3	GCGCAGTTTTGCCAGAGGGCGC	seq29	GCGCATACCAACGGTGCTGCGC
seq4	GCGCGAGGCACTGTTTACGCGC	seq30	GCGCTGCTCTATATCCCTGCGC
seq5	GCGCTTACTAATCTCATTGCGC	seq31	GCGCCCCTGGGCTGGAAGGCGC
seq6	GCGCCATTATACAAATCAGCGC	seq32	GCGCGAAGCTCCTGTGGTGCGC
seq7	GCGCATCAATCGCATCTTGCGC	seq33	GCGCTGGTGAACGTCTAGGCGC
seq8	GCGCTCTTAGAAGGGCATGCGC	seq34	GCGCCTAGCTGAAAGTCTGCGC
seq9	GCGCGCATTCAAGCCTGAGCGC	seq35	GCGCGTCTCCAAGGACGCGCGC
seq10	GCGCCTGAGCGCGGTTGAGCGC	seq36	GCGCACGCCTATCATGCTGCGC
seq11	GCGCTTGAGTTCGTCAAAGCGC	seq37	GCGCTGCTTACCTAACCAGCGC
seq12	GCGCCAAACCCCAATTACGCGC	seq38	GCGCACCAGTTGCGCCCGGCGC
seq13	GCGCTTACACGCGAGGGGGCGC	seq39	GCGCCCCGGAACCTTTAGGCGC
seq14	GCGCGGGGCCGTGCGTAGGCGC	seq40	GCGCTTAGCAAGTAGCGAGCGC
seq15	GCGCGTAGTGTCGCCGGTGCGC	seq41	GCGCGCGAATTTAATAAGGCGC
seq16	GCGCCGGTACGACTGATCGCGC	seq42	GCGCTAAGGCCATCCATGGCGC
seq17	GCGCGATCCTACAGAATCGCGC	seq43	GCGCCATGTGTTCTCGATGCGC
seq18	GCGCAATCCGCCAAAGACGCGC	seq44	GCGCCGATGACTAGGGTAGCGC
seq19	GCGCAGACATTGCAGCAGGCGC	seq45	GCGCGGTAGATACTCCCGGCGC
seq20	GCGCGCAGGTCGAAATGGGCGC	seq46	GCGCCCCGCTGTCAGACCGCGC
seq21	GCGCATGGGACTCGGACAGCGC	seq47	GCGCGACCCACCTCACGAGCGC
seq22	GCGCGACAACATATTCCTGCGC	seq48	GCGCACGATCTGCGGCAGGCGC
seq23	GCGCTCCTCCGTCGGGTGGCGC	seq49	GCGCGCAGTACATCACTTGCGC
seq24	GCGCGGTGTGACCATAACGCGC	seq50	GCGCACTTAACAATACGTGCGC
seq25	GCGCTAACTTCGGCTCGTGCGC	seq51	GCGCACGTGGACCGATAAGCGC
seq26	GCGCTCGTTACGGGGGAAGCGC	seq52	GCGCATAAAATATACGTAGCGC

Table S2. Simulated oligomers comprising all 512 unique RNA pentamers (set52, RNA).

RNA	5'--------------------3'	RNA	5'--------------------3'
seq1	GCGCAAAAUAGCCACUCUGCGC	seq27	GCGCGGAAACGCUUUUUCGCGC
seq2	GCGCCUCUCUUCAUAGUUGCGC	seq28	GCGCUUUCUUGUGCAUACGCGC
seq3	GCGCAGUUUUGCCAGAGGGCGC	seq29	GCGCAUACCAACGGUGCUGCGC
seq4	GCGCGAGGCACUGUUUACGCGC	seq30	GCGCUGCUCUAUAUCCCUGCGC
seq5	GCGCUUACUAAUCUCAUUGCGC	seq31	GCGCCCCUGGGCUGGAAGGCGC
seq6	GCGCCAUUAUACAAAUCAGCGC	seq32	GCGCGAAGCUCCUGUGGUGCGC
seq7	GCGCAUCAAUCGCAUCUUGCGC	seq33	GCGCUGGUGAACGUCUAGGCGC
seq8	GCGCUCUUAGAAGGGCAUGCGC	seq34	GCGCCUAGCUGAAAGUCUGCGC
seq9	GCGCGCAUUCAAGCCUGAGCGC	seq35	GCGCGUCUCCAAGGACGCGCGC
seq10	GCGCCUGAGCGCGGUUGAGCGC	seq36	GCGCACGCCUAUCAUGCUGCGC
seq11	GCGCUUGAGUUCGUCAAAGCGC	seq37	GCGCUGCUUACCUAACCAGCGC
seq12	GCGCCAAACCCCAAUUACGCGC	seq38	GCGCACCAGUUGCGCCCGGCGC
seq13	GCGCUUACACGCGAGGGGGCGC	seq39	GCGCCCCGGAACCUUUAGGCGC
seq14	GCGCGGGGCCGUGCGUAGGCGC	seq40	GCGCUUAGCAAGUAGCGAGCGC
seq15	GCGCGUAGUGUCGCCGGUGCGC	seq41	GCGCGCGAAUUUAAUAAGGCGC
seq16	GCGCCGGUACGACUGAUCGCGC	seq42	GCGCUAAGGCCAUCCAUGGCGC
seq17	GCGCGAUCCUACAGAAUCGCGC	seq43	GCGCCAUGUGUUCUCGAUGCGC
seq18	GCGCAAUCCGCCAAAGACGCGC	seq44	GCGCCGAUGACUAGGGUAGCGC
seq19	GCGCAGACAUUGCAGCAGGCGC	seq45	GCGCGGUAGAUACUCCCGGCGC
seq20	GCGCGCAGGUCGAAAUGGGCGC	seq46	GCGCCCCGCUGUCAGACCGCGC
seq21	GCGCAUGGGACUCGGACAGCGC	seq47	GCGCGACCCACCUCACGAGCGC
seq22	GCGCGACAACAUAUUCCUGCGC	seq48	GCGCACGAUCUGCGGCAGGCGC
seq23	GCGCUCCUCCGUCGGGUGGCGC	seq49	GCGCGCAGUACAUCACUUGCGC
seq24	GCGCGGUGUGACCAUAACGCGC	seq50	GCGCACUUAACAAUACGUGCGC
seq25	GCGCUAACUUCGGCUCGUGCGC	seq51	GCGCACGUGGACCGAUAAGCGC
seq26	GCGCUCGUUACGGGGGAAGCGC	seq52	GCGCAUAAAAUAUACGUAGCGC

Table S3. DNA and RNA sequences containing all tetramers (set14).

DNA	5'-------------------3'	RNA	5'-------------------3'
seq1	GCGCGGACGTTCAGCGACGCGC	seq1	GCGCGGACGUUCAGCGACGCGC
seq2	GCGCGCCCTGCATACAGTGCGC	seq2	GCGCGCCCUGCAUACAGUGCGC
seq3	GCGCTATATCTAGCTTGAGCGC	seq3	GCGCUAUAUCUAGCUUGAGCGC
seq4	GCGCGGAGTTGTCATGTTGCGC	seq4	GCGCGGAGUUGUCAUGUUGCGC
seq5	GCGCTCGAGCACTTAACCGCGC	seq5	GCGCUCGAGCACUUAACCGCGC
seq6	GCGCTGATCGGTGAGGATGCGC	seq6	GCGCUGAUCGGUGAGGAUGCGC
seq7	GCGCGACTACGAATGGTCGCGC	seq7	GCGCGACUACGAAUGGUCGCGC
seq8	GCGCGAGAAATTGCCTAAGCGC	seq8	GCGCGAGAAAUUGCCUAAGCGC
seq9	GCGCGCGCGGAAGGTAATGCGC	seq9	GCGCGCGCGGAAGGUAAUGCGC
seq10	GCGCGGCGTGGCCGGGAGGCGC	seq10	GCGCGGCGUGGCCGGGAGGCGC
seq11	GCGCGTACTGGGTCTTTAGCGC	seq11	GCGCGUACUGGGUCUUUAGCGC
seq12	GCGCTCCAAAACGGGGCTGCGC	seq12	GCGCUCCAAAACGGGGCUGCGC
seq13	GCGCAATAATCTGTGTCGGCGC	seq13	GCGCAAUAAUCUGUGUCGGCGC
seq14	GCGCATAGAGATGCGCGCGCGC	seq14	GCGCAUAGAGAUGCGCGCGCGC

Table S4. Absolute errors of coordinate means computed for the first and the second half of a filtered trajectory with respect to values for the whole trajectory. The values shown are averaged over all the 107 DNA and RNA trajectories.

	**DNA**	**RNA**
Shear [Å]	0.0013	0.0010
Stretch [Å]	0.0003	0.0004
Stagger [Å]	0.0018	0.0021
Buckle [°]	0.0857	0.0578
Propeller [°]	0.0581	0.0544
Opening [°]	0.0197	0.0229
Shift [Å]	0.0082	0.0040
Slide [Å]	0.0060	0.0037
Rise [Å]	0.0019	0.0016
Tilt [°]	0.0260	0.0202
Roll [°]	0.0574	0.0583
Twist [°]	0.0628	0.0272

Table S5. Relative errors of coordinate stiffnesses computed for the first and the second half of a filtered trajectory with respect to values for the whole trajectory. The values shown are averaged over all the 107 DNA and RNA trajectories.

	**DNA**	**RNA**
Shear	0.31%	0.40%
Stretch	0.35%	0.50%
Stagger	0.34%	0.44%
Buckle	0.56%	0.50%
Propeller	0.48%	0.61%
Opening	0.37%	0.50%
Shift	0.83%	0.69%
Slide	0.83%	0.68%
Rise	0.46%	0.49%
Tilt	0.36%	0.46%
Roll	0.64%	0.68%
Twist	0.93%	0.96%

Table S6. Relative errors of global stiffness constants for the first and the second half of a filtered trajectory with respect to values for the whole trajectory. The values shown are averaged over all the 107 DNA and RNA trajectories (set 107), and over the 52 trajectories of set52.

	set107 DNA	set107 RNA	set52 DNA	set52 RNA
Y_{1D} [pN]	0.011	0.025	0.020	0.015
C_{1D} [nm]	0.012	0.021	0.014	0.011
$l_{tw}/2$ [nm]	0.015	0.025	0.020	0.016
k [nm/turn]	0.024	0.055	0.028	0.041
l_d [nm]	0.024	0.037	0.028	0.023

Table S7. Averaged absolute differences between coordinate means for the central step of duplicate hexamers.

	DNA	RNA
Shift [Å]	0.0507	0.0170
Slide [Å]	0.0438	0.0238
Rise [Å]	0.0281	0.0189
Tilt [°]	0.2185	0.1783
Roll [°]	0.2570	0.2841
Twist [°]	0.4670	0.1450

Table S8. Averaged relative differences between coordinate stiffnesses for the central step of duplicate hexamers.

	DNA	RNA
Shift	4.40%	1.99%
Slide	4.46%	1.88%
Rise	2.88%	1.75%
Tilt	1.32%	1.13%
Roll	2.14%	1.81%
Twist	5.56%	1.51%

Table S9. Means and standard deviations of equilibrium coordinates for all the hexamers with the indicated step in the middle. Data for RNA duplexes.

Middle dimer	Shift [Å]	Slide [Å]	Rise [Å]	Tilt [°]	Roll [°]	Twist [°]
**CG**	0	-1.67±0.05	3.47±0.10	0	11.08±1.10	30.72±0.31
**CA**	-0.01±0.04	-1.49±0.03	3.38±0.08	0.52±0.78	12.86±1.18	31.34±0.31
**UA**	0	-1.40±0.04	3.32±0.09	0	14.93±1.21	31.68±0.32
**AG**	-0.05±0.05	-1.73±0.04	3.29±0.06	-0.69±0.73	8.35±0.76	28.94±0.30
**GG**	0.10±0.04	-1.87±0.03	3.36±0.06	0.16±0.55	9.31±0.76	30.34±0.32
**AA**	-0.07±0.04	-1.55±0.04	3.25±0.06	-0.57±0.53	8.34±0.98	28.85±0.32
**GA**	0.09±0.05	-1.62±0.04	3.25±0.07	-0.34±0.50	7.92±1.01	30.38±0.32
**AU**	0	-1.50±0.06	3.19±0.03	0	6.14±0.55	28.79±0.32
**AC**	-0.12±0.05	-1.67±0.10	3.26±0.03	-0.31±0.33	5.79±0.56	29.81±0.39
**GC**	0	-1.76±0.09	3.25±0.02	0	4.00±0.29	30.82±0.38
**Avg**	-0.01±0.07	-1.63±0.14	3.30±0.08	-0.12±0.36	8.87±3.33	30.17±1.04

Table S10. Errors on the equilibrium intra-base pair coordinates and minor groove widths predicted by the heptanucleotide model, compared to the actual MD values from the set52 validation dataset.

	**DNA**	**RNA**
Shear [Å]	0.005	0.005
Stretch [Å]	0.002	0.002
Stagger [Å]	0.017	0.014
Buckle [°]	0.983	0.581
Propeller [°]	0.623	0.392
Opening [°]	0.108	0.080
Minor g. width [Å]	0.162	0.046

Table S11. Errors on the equilibrium inter-base pair and helical coordinates, and major groove widths, predicted by the hexanucleotide model, in comparison to the actual MD values from the set52 validation dataset.

	**DNA**	**RNA**
Shift [Å]	0.042	0.016
Slide [Å]	0.038	0.017
Rise [Å]	0.025	0.017
Tilt [°]	0.186	0.154
Roll [°]	0.251	0.248
Twist [°]	0.457	0.141
X-disp [Å]	0.082	0.032
Y-disp [Å]	0.067	0.034
h-Rise [Å]	0.024	0.016
Inclination [°]	0.419	0.395
Tip [°]	0.315	0.280
h-Twist [°]	0.448	0.181
Major g. width [Å]	0.264	0.106

Table S12. Relative model errors on coordinate stiffness constants compared to the validation set s52.

	DNA	RNA
Shear	4.17 %	1.99 %
Stretch	3.70 %	1.65 %
Stagger	2.50 %	1.56 %
Buckle	1.36 %	1.03 %
Propeller	2.55 %	1.58 %
Opening	4.58 %	1.85 %
Minor g.	1.35 %	0.27 %
Shift	0.72 %	0.63 %
Slide	0.95 %	0.78 %
Rise	1.20 %	0.99 %
Tilt	3.40 %	1.42 %
Roll	2.36 %	1.41 %
Twist	1.20 %	0.91 %
Major g.	1.44 %	0.59 %

Table S13. Deformation energy of A-tracts and the control sequence threaded through the nucleosome. Averages and standard deviations of energies for the range of dyad positions indicated.

Sequence	Range of nucleosome dyad positions (bp)	Deformation energy (kcal/mol)
Control	74 - 328	242 ± 15
A_{21}	138 - 264	263 ± 14
A_{41}	148 - 254	281 ± 13
A_{61}	158 - 244	301 ± 14

References

1. Orenstein, Y. and Shamir, R. (2013) Design of shortest double-stranded DNA sequences covering all k-mers with applications to protein-binding microarrays and synthetic enhancers. *Bioinformatics*, **29**, i71-i79.

2. Zgarbova, M., Jurecka, P., Lankas, F., Cheatham III, T.E., Sponer, J. and Otyepka, M. (2017) Influence of BII backbone substates on DNA twist: A unified view and comparison of simulation and experiment for all 136 distinct tetranucleotide sequences. *J. Chem. Inf. Model.*, **57**, 275-287.

3. Trifonov, E.N., Tan, R.K.Z. and Harvey, S.C. (1988) In Olson, W. K., Sarma, M. H., Sarma, R. H. and Sundaralingam, M. (eds.), *Structure and Expression*. Adenine Press, Inc., Albany, NY, pp. 243-254.

4. Drsata, T., Perez, A., Orozco, M., Morozov, A.V., Sponer, J. and Lankas, F. (2013) Structure, stiffness and substates of the Dickerson-Drew dodecamer. *J. Chem. Theory Comput.*, **9**, 707-721.
